# Efficient synthesis of α-galactosylceramide and its C-6 modified analogs

**DOI:** 10.3389/fchem.2022.1039731

**Published:** 2022-11-25

**Authors:** Huiting Li, Hongzhao Mao, Chao Chen, Ying Xu, Shuai Meng, Tiantian Sun, Chengli Zong

**Affiliations:** ^1^ Key Laboratory of Tropical Biological Resources of Ministry of Education, School of Pharmaceutical Sciences, College of Marine Science, Hainan University, Haikou, China; ^2^ Anyikang Co., LTD., Shanghai, China

**Keywords:** α-galactosylceramide, glycosylation, orthogonal protection strategy, click chemistry, analogs

## Abstract

The synthesis of α-galactosylceramide (KRN7000) and its C-6 modified analogs remains a challenge due to the difficult α-1,2-*cis*-glycosidic bond. A non-participating benzyl (Bn) protecting group has been commonly used to favor the α-glycosylation product. Here, we report the α-selective glycosylation by using a bulky 4,6-O-di-*tert*-butylsilylene (DTBS) galactosyl donor, regardless of the 2-benzoyl (Bz) participating group. Compared with Bn, Bz groups can be selectively removed in basic conditions without impacting the C-6 azide modification. The azide has the potential for clicking with alkyne or being easily transformed to other functional groups.

## Graphical Abstract

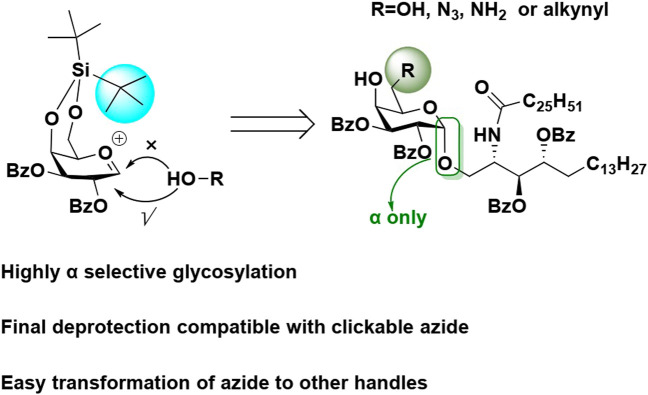

## 1 Introduction

α-Galactosylceramide or KRN7000 originated from the marine sponge *Agelas mauritiana* (M Morita 1995). It is an effective activator of invariant natural killer T (iNKT) cells by forming a ternary complex with CD1d and T-cell receptor (van der Vliet HJ 2001). iNKT cells can bridge the innate and adaptive immunity (Van Kaer et al., 2011). On one side, these cells mostly resemble cells of the innate immune system: they express semi-invariant T-cell receptors that can interact with a limited set of lipid and glycolipid antigens, resembling the pattern recognition receptors of the innate immune system (Borg et al., 2007). Also, contrary to the slow adaptive immune cells, activated iNKT cells can rapidly prime other effectors and cannot develop immunological memory. On the other side, activated iNKT cells can produce both Th-1- and Th-2-type cytokines that can jump-start and modulate an adaptive immune response.

KRN7000 is an extremely potent adjuvant to activate iNKT cells, which plays a unique role in the development of various types of vaccines. It has been used as a vaccine adjuvant against cancer (Chen et al., 2020), bacteria (Sun et al., 2022), and virus (Akache et al., 2022) (Carreno et al., 2014). Compared with other adjuvants, it has some unique characteristics. Activating iNKT cells too strongly may be detrimental for live attenuated virus due to the fast clearance by iNKT cell-mediated immune response before they had an opportunity to induce vaccine-specific immunity (Li et al., 2011; Schneiders et al., 2011). Also, repeated injection of KRN7000 can hyper-energize and inactivate iNKT cells (Parekh et al., 2005). Because the antigen-presenting molecule CD1d is non-polymorphic, KRN7000-activated iNKT cells can provide a universal form of T-cell help for a vaccine in humans with diverse haplotypes (Li et al., 2022).

Traditionally, adjuvants are physically mixed with an antigen to enhance the vaccines’ immunogenicity (Das and Mukhopadhyay, 2020). Recently, the covalent conjugation of an antigen and a synthetic adjuvant is becoming a promising strategy to stimulate the innate immune system (K H Wiesmüller 1991; Huang et al., 2020; Liu et al., 2020; Massena et al., 2021; Yin et al., 2021). It has been established that the CD1d-KRN7000 interaction tolerates the modification at C-6 of KRN7000 (Chennamadhavuni et al., 2018; Kaplonek et al., 2018).

Efficient synthesis of C-6-modified KRN7000 analogs remains a challenge. Multiple studies have confirmed that only the α-1,2-*cis*-anomer of KRN7000 is active (Yin et al., 2017; Chen et al., 2019). The formation of a 1,2-*trans*-β-glycosidic bond is relatively easy by using a neighboring group participation effect (Huang et al., 2015; Fang et al., 2016). A non-participating Bn group is usually employed at the C-2 position to favor a thermodynamically stable α-product. The final hydrogenation to remove the Bn protecting groups is not compatible with conventional un-saturated clickable handles, such as the azide or alkyne. They must be introduced after Bn removal, but we found de-protected KRN7000 demonstrated poor solubility in common organic solvents, making these manipulations difficult.

With an interest to develop a robust approach to synthesize C-6-modified KRN7000, in this study, we describe an efficient procedure for the synthesis of KRN7000 and its analogs based on 4,6-di-*tert*-butylsilylene (DTBS), and C-2 and C-3 Bz protected donors. In this approach, the final deprotection is compatible with the C-6 azide modification and can be easily transformed to other reactive groups such as amines and alkynes.

## 2 Materials and methods

### 2.1 Chemistry

#### 2.2.1 General considerations

Unless otherwise noted, all materials and solvents were used as received from Adamas-beta® without further purification. NMR spectra were recorded on the Varian Mercury 500-MHz spectrometers. Chemical shifts are reported in parts per million (ppm) relative to MeOH-*d*_*4*_, CDCl_3_, or TMS signature chemical shifts. NMR data are presented as follows: chemical shift, multiplicity (s = singlet, d = doublet, t = triplet, dd = doublet of doublet, and m = multiplet and/or multiple resonances), coupling constant in hertz (Hz), and integration. ^13^C data were derived from the HSQC spectrum. All NMR signals were assigned on the basis of ^1^H NMR, COSY, and HSQC experiments. Mass spectra were recorded on a Shimadzu LCMS-IT-TOF mass spectrometer or a Bruker IT-TOF mass spectrometer. TLC analysis was performed on silica gel 60 F254 (Huang Hai Inc.) with detection by UV absorption (254 nm) when applicable and by spraying with a solution of (NH_4_)_6_Mo_7_O_24_·H_2_O (25 g/L) in 5% sulfuric acid in ethanol, followed by charring. All reactions were carried out under an argon atmosphere.

*(2S,3S,4R)-2-Azido-1-(((2,3-dimethylbutan-2-yl)dimethylsilyl)oxy) octadecane-3,4-diol (* ***11*** *).* Compound **10** (2.2 g) and imidazole (2 equiv, 872 mg) were dissolved in anhydrous DMF, followed by dropwise addition of TDSCl (1.2 equiv, 1.51 ml) at 0°C. Stirring was continued until TLC (hexane/EtOAc, 1/1, v/v) indicated disappearance of starting material (∼30 min) and then quenched with methanol. The reaction mixture was diluted with EtOAc and then washed with NaHCO_3_ and brine. The organic layer was dried (Na_2_SO_4_) and filtered. The filtrate was concentrated under reduced pressure, and the residue was purified by silica gel column chromatography using a gradient of hexanes and EtOAc (from 50/10 to 30/10, v/v) to afford compound **11** (2.292 g, 60%). HRMS (ESI^+^) calcd for C_26_H_55_N_3_NaO_3_Si (M^+^+Na) 508.4013, found 508.4021. ^1^H NMR (500 MHz, CDCl_3_) δ 4.00 (dd, *J* = 10.7, 4.4 Hz, 1H, H-1^A^), 3.91 (dd, J = 10.5, 4.8 Hz, 1H, H-1^B^), 3.70 (S, 2H, H-3, H-4), 3.52 (d, *J* = 5.0 Hz, 1H, H-2), 1.19–1.40 (m, 27H, -C_13_ *H*_26_-CH_3_, TDS-C*H*), 0.93–0.81 (m, 15H, -C_13_H_26_-C*H*_3_, TDS -C*H*_3_), and 0.16 (s, 6H, -Si-C*H*_3_). ^13^C NMR (126 MHz, CDCl_3_) δ 74.71, 72.51, 63.13, 63.13, 62.99, 62.55, 33.98, 32.07, 29.58, 25.62, 24.74, 22.55, 22.55, 20.06, 18.44, 18.30, and 14.05.

*(2S,3S,4R)-2-azido-1-hydroxyoctadecane-3,4-diyl dibenzoate (* ***7*** *).* DAMP (0.3 equiv, 204 mg) was added to a solution of **11** (2.7 g) in anhydrous pyridine (28 ml) and then cooled to 0°C. BzCl (8 equiv, 5.17 ml) was added drop-wise. Stirring was continued until TLC (hexane/EtOAc, 11/1, v/v) indicated disappearance of starting material (∼12 h). CH_3_OH was added, and stirring was continued for 30 min. The mixture was concentrated under reduced pressure. The residue was diluted with CH_2_Cl_2_, washed with NaHCO_3_ and brine, dried (Na_2_SO_4_), and filtered. The filtrate was concentrated under reduced pressure, and the residue was purified by silica gel column chromatography using a gradient of hexanes and EtOAc to afford a pure product (3.43 g, 89%). The pure product (3.8 g) was dissolved in anhydrous THF, and HF-pyridine (20 equiv, 10 ml) was added drop-wise at 50°C. The reaction mixture was stirred at 50°C for 12 h until TLC analysis (hexane/EtOAc, 1/1, v/v) indicated completion of the reaction and then quenched with saturated NaHCO_3._ The mixture was concentrated under reduced pressure, and then, the residue was diluted with EtOAc and washed with brine. The organic layer was collected, dried (Na_2_SO_4_), and filtered. The filtrate was concentrated under reduced pressure, and the residue was purified by silica gel column chromatography using a gradient of hexanes and EtOAc (from 15/1 to 12/1, v/v) to afford compound **7** (2.53 g, 84%). HRMS (ESI^+^) calcd for C_32_H_45_N_3_NaO_5_ (M^+^+Na) 574.3359, found 574.3367. ^1^H NMR (500 MHz, CDCl_3_) δ 8.07–7.37 (m, 10H, C*H* aromatics), 5.60–5.49 (m, 2H, H-3, H-4), 3.99 (d, *J* = 8.9 Hz, 1H, H-1^A^), 3.85–3.76 (m, 2H, H-1^B^, H-2), 2.00–1.82 (m, 2H, -C*H*_2_-C_12_H_24_-CH_3_), 1.23 (d, *J* = 10.2 Hz, 24H, -CH_2_-C_12_ *H*_24_-CH_3_), and 0.87 (t, *J* = 6.9 Hz, 3H, -CH_2_-C_12_H_24_-C*H*_3_). ^13^C NMR (126 MHz, CDCl_3_) δ 133.67, 133.19, 130.01, 129.77, 129.52, 128.55, 128.30, 72.87, 63.10, 61.64, 29.40, 29.40, 25.50, 22.57, and 13.77.

*p-Tolyl 2,3-di-O-benzoyl-4,6-di-O-di-tert-butylsilylidene-1-thio-D-galactopyranoside (* ***6*** *).* Compound **6** was prepared according to previously reported procedures. HRMS (ESI^+^) calcd for C_35_H_42_O_7_SNaSi (M^+^+Na) 657.2421, found 657.2429. ^1^H NMR (500 MHz, CDCl_3_) δ 8.14–7.35 (m, 14H, C*H* aromatics), 5.91 (t, *J* = 10.0 Hz, 1H, H-2), 5.19 (d, *J* = 9.8 Hz, 1H, H-3), 4.93–4.83 (m, 2H, H-1, H-4), 4.30 (q, *J* = 10.0 Hz, 2H, H-6), 3.62 (s, 1H, H-5), 2.31 (s, 3H, C*H*_*3*_ of STol), 1.15 (s, 9H, C*H*_3_ of DTBS), and 0.95 (s, 9H, C*H*_3_ of DTBS). ^13^C NMR (126 MHz, CDCl_3_) δ 133.74, 133.25, 133.25, 133.25, 130.32, 130.07, 129.83, 129.58, 128.36, 128.36, 128.36, 87.83, 87.83, 75.37, 74.88, 70.25, 68.05, 68.05, 67.07, 27.27, 27.27, 27.27, 27.27, and 20.92.

*ɑ-D-Galactopyranoside,(2S,3S,4R)-2-azido-3,4-bis(benzoyloxy) octadecyl 4, 6-O-[bis(1,1-dimethylethyl)silylene]-,2,3-dibenzoate (* ***5*** *)*.

#### 2.2.2 Thiol donor

Compounds **7** (1 equiv, 890 mg) and **6** (1.5 equiv, 1.54 g) were dissolved in CH_2_Cl_2_, dried by co-evaporation, pumped for about 1 h on cold nitrile, and then dissolved in anhydrous CH_2_Cl_2_. Freshly activated powdered molecular sieves (4 Å) were added, and the mixture was stirred for 10 min at room temperature, and then, NIS(2 equiv, 1.15 g) was added and cooled to 0°C. TfOH (0.3 equiv, 42.8 ul) was added, and the resulting reaction mixture was stirred for 6 h at room temperature and then quenched by the addition of TEA. The mixture was concentrated under reduced pressure, and the residue was purified by silica gel column chromatography using a gradient of hexanes and EtOAc (from 10/1 to 80/10, v/v) to afford **5** (957 mg, 56%).

#### 2.2.3 Imidate donor

Compound **7** (100 mg) was dissolved in 2.7 ml of acetone and 300 μL of water cooled at 0°C, and NBS was added (2 equiv, 56 mg). The resulting reaction mixture was stirred for 30 min at room temperature before being quenched with saturated NaHCO_3_ and saturated Na_2_S_2_O_3_ solution. Acetone was removed under reduced pressure, and the remaining aqueous mixture was extracted with EtOAc. Combined extracts were sequentially washed with water and brine. The organic layer was collected, dried (Na_2_SO_4_), and filtered. The filtrate was concentrated under reduced pressure, and the residue was purified by silica gel column chromatography using a gradient of hexanes and EtOAc (from 60/10 to 40/10, v/v) to afford pure product (55 mg, 66%). The pure product (86 mg) and 2,2,2-trifluoro-N-phenylethanimidoyl chloride (1.5 equiv, 39.6 ul) were dissolved in CH_2_Cl_2_ and cooled to 0°C; then, NaH (1.5 equiv, 9.76 mg) was added. The resulting reaction mixture was stirred for 30 min at room temperature. The mixture was concentrated under reduced pressure, and the residue was purified by silica gel column chromatography using a gradient of hexanes and EtOAc (from 10/1 to 15/1, v/v) to afford N-phenyl donor (102 mg, 90%). Compound **6** (1 equiv, 65 mg) and N-phenyl donor (1.5 equiv, 124 mg) were dissolved in CH_2_Cl_2_, dried by co-evaporation, pumped for about 1 h on cold nitrile, and then dissolved in anhydrous CH_2_Cl_2_. Freshly activated powdered molecular sieves (4 Å) were added, and the mixture was stirred for 10 min at room temperature and then cooled to 0°C. TfOH (0.3 equiv, 42.8 ul) was added, and the resulting reaction mixture was stirred for 40 min at 0°C and then quenched by the addition of TEA. The mixture was concentrated under reduced pressure, and the residue was purified by silica gel column chromatography using a gradient of hexanes and EtOAc (from 10/1 to 80/10, v/v) to afford **5** (78 mg, 62%). HRMS (ESI^+^) calcd for C_60_H_79_N_3_NaO_12_Si (M^+^+Na) 1084.5433, found 1084.5425. ^1^H NMR (500 MHz, CDCl_3_) δ 8.03–7.27 (m, 20H, C*H* aromatics), 5.79 (d, *J* = 10.8 Hz, 1H, H-2), 5.54 (d, *J* = 10.5 Hz, 1H, H-3), 5.48 (d, *J* = 5.9 Hz, 2H, H-9, H-10), 5.31 (d, *J* = 3.6 Hz, 1H, H-1), 4.87 (s, 1H, H-4), 4.29–4.14 (m, 2H, H-6), 3.96 (s, 1H, H-8), 3.88 (s, 1H, H-5), 3.67 (t, *J* = 9.5 Hz, 2H, H-7), 1.80 (s, 2H, -C*H*_2_-C_12_H_24_-CH_3_), 1.27–1.09 (m, 24H, -CH_2_-C_12_ *H*_24_-CH_3_), and 0.87 (t, *J* = 6.6 Hz, 3H, -CH_2_-C_12_H_24_-C*H*_3_). ^13^C NMR (126 MHz, CDCl_3_) δ 133.25, 133.01, 132.76, 129.59, 128.37, 128.12, 128.12, 97.11, 72.69, 70.74, 70.49, 67.89, 67.56, 67.07, 66.59, 60.97, 29.44, 29.22, 27.03, and 26.78.

*[(1S,2S,3R)-1-[[[2,3-Bis-O-(Dibenzoyloxy)-ɑ-D-galactopyranosyl]oxy]methyl]-2,3-bis(*Dibenzoyloxy*)heptadecyl]hexacosanamide (* ***14*** *).* Compound **5** (1.5 g) was dissolved in CH_2_Cl_2_/MeOH (1:1, 50 ml) and stirred with a tablespoon Pd/C and 1M HCl (1.2 equiv) under H_2_ atmosphere at room temperature for 8 h. After the disappearance of starting material monitored by TLC, the reaction mixture was filtered through a 0.22-um nylon microporous membrane. The mixture was concentrated under reduced pressure, and the residue was purified by silica gel column chromatography using a gradient of hexanes and EtOAc (from 60/10 to 30/10, v/v) to give a pure product. Equimolar hydrochloric acid was added when the pure product was concentrated (1.16 g, 80%). Ceric acid (1.5 equiv, 330 mg), EDC (7 equiv, 734 mg), and HOBT (7 equiv, 524 mg) were dissolved in anhydrous THF/DMF (1:1, 10 ml) and then cooled to 0°C. DAMP (0.1 equiv, 6.77 mg) was added, and the resulting reaction mixture was stirred for 30 min at room temperature. The pure product (1 equiv, 574 mg) was dissolved in anhydrous THF (5 ml), DIPEA (3 equiv, 275 ul) was added, the step 1 reaction mixture was added dropwise into the step 2 reaction mixture, and the reaction flask in step 1 was washed with 2 ml of THF, stirring the reaction overnight at room temperature. Then, it was quenched with water and concentrated under reduced pressure. The residue was diluted with CH_2_Cl_2_, washed with NaHCO_3_, water, and brine. The organic layer was collected, dried (Na_2_SO_4_), and filtered, and the filtrate was concentrated under reduced pressure. The residue (784 mg) was diluted in anhydrous THF (15 ml). HF-Pyridine (20 equiv, 998 ul) was added dropwise, and the resulting reaction mixture was stirred for 4 h at room temperature and then quenched with NaHCO_3_. The mixture was concentrated under reduced pressure until only the aqueous layer remained. The residue was diluted with EtOAc and washed with brine. The organic layer was collected, dried (Na_2_SO_4_), and filtered. The filtrate was concentrated under reduced pressure, and the residue was purified by silica gel column chromatography using a gradient of hexanes and EtOAc (from 60/10 to 30/10, v/v) to afford **14** (353 mg, 50% for two steps). HRMS (ESI^+^) calcd for C_78_H_115_NNaO_13_ (M^+^+Na) 1296.8368, found 1296.8362. ^1^H NMR (500 MHz, CDCl_3_) δ 8.03–7.21 (m, 20H, C*H* aromatics), 7.26 (s, 1H, -N*H*), 5.81 (d, *J* = 9.9 Hz, 1H, H-9), 5.59 (s, 2H, H-2, H-3), 5.27–5.14 (m, 2H, H-1, H-10), 4.56 (t, *J* = 10.3 Hz, 1H, H-8), 4.37 (s, 1H, H-4), 4.33 (t, *J* = 5.9 Hz, 1H, H-5), 4.06 (dd, *J* = 12.6, 3.5 Hz, 1H, H-7^A^), 4.01 (s, 2H, H-6), 3.60 (d, *J* = 12.4 Hz, 1H, H-7^B^), 2.31 (t, *J* = 7.5 Hz, 2H, -C*H*_2_-C_22_H_44_-CH_3_), 1.92 (d, *J* = 8.5 Hz, 2H, -C*H*_2_-C_12_H_24_-CH_3_), 1.17 (m, 68H, -CH_2_-C_12_ *H*_24_-CH_3_, -CH_2_-C_22_ *H*_44_-CH_3_), and 0.87 (t, *J* = 6.9 Hz, 6H, -CH_2_-C_12_H_24_-C*H*_3_, -CH_2_-C_22_H_44_-C*H*_3_). ^13^C NMR (126 MHz, CDCl_3_) δ 133.43, 133.19, 133.19, 133.19, 133.19, 129.77, 129.77, 129.77, 129.77, 129.77, 129.52, 129.52, 128.55, 128.30, 128.30, 128.30, 128.30, 128.30, 128.30, 99.49, 74.58, 72.63, 71.65, 71.16, 70.92, 69.21, 68.72, 64.12, 62.37, 48.69, 36.49, 29.40, 29.40, 27.45, and 13.77.

α-Galactosylceramide. Compound **14** (1 equiv, 100 mg) was dissolved in anhydrous MeOH/DCM (1:1, 2 ml), and 10 mg of solid sodium metal was added and dissolved in 1 ml of anhydrous methanol; the resulting reaction mixture was stirred for 4 h at room temperature. Cationic resin was added to neutralize, filtered, and then concentrated *in vacuo*. The resulting residue was purified by column chromatography on silica gel EtOAc/pyridine to afford KRN7000 (48.5 mg, 72%). HRMS (ESI^+^) calcd for C_50_H_99_NNaO_9_ [M + Na]^+^ 880.7320, found 880.7329. ^1^H NMR (500 MHz, C_5_H_5_N) δ 8.56 (d, J = 8.4 Hz, 1H, -N*H*), 5.60 (d, *J* = 4.0 Hz, 1H, H-1), 5.29 (d, *J* = 8.7 Hz, 1H, H-8), 4.69 (q, *J* = 5.5 Hz, 2H, H-2, H-7^A^), 4.60–4.50 (m, 2H, H-4, H-5), 4.49–4.38 (m, 3H, H-3, H-6, H-7^B^), 4.34 (d, *J* = 8.4 Hz, 2H, H-9, H-10), 1.93 (s, 2H, -C*H*_2_-C_22_H_44_-CH_3_), 1.83 (dd, *J* = 9.5, 5.5 Hz, 2H, -C*H*_2_-C_12_H_24_-CH_3_), 1.75–1.07 (m, 68H, -CH_2_-C_12_ *H*_24_-CH_3_, -CH_2_-C_22_ *H*_44_-CH_3_), and 0.88 (t, *J* = 6.6 Hz, 6H, -CH_2_-C_12_H_24_-C*H*_3_, -CH_2_-C_22_H_44_-C*H*_3_). ^13^C NMR (126 MHz, C_5_H_5_N) δ 101.29, 76.39, 72.72, 72.24, 71.26, 70.77, 70.04, 68.33, 62.47, 51.23, 34.14, 26.08, 22.42, and 13.87.

*[(1S,2S,3R)-1-[[[6-O-[(4-Methylphenyl)sulfonyl]-2,3-bis-O-(Dibenzoyloxy)-ɑ-D-galactopyranosyl]oxy]methyl]-2,3-bis(Dibenzoyloxy)heptadecyl]hexacosanamide (* ***15*** *).* Compound **14** (1.0 equiv, 250 mg, 196 umol) was dissolved in anhydrous CH_2_Cl_2_ (8 ml), and TsCl (3 equiv, 112 mg, 588 umol) was added, followed by Et_3_N (137 ul, 5 equiv, 981 umol) at 0°C. The reaction mixture was raised up to room temperature and stirred for 5 h. Saturated aqueous NH_4_Cl was slowly added to neutralize. Two layers were separated, and the aqueous layer was extracted with CH_2_Cl_2_. The organic extracts were combined and dried over anhydrous Na_2_SO_4_, and then concentrated *in vacuo*. The resulting residue was purified by column chromatography on silica gel hexanes/EtOAc (from 50/10 to 30/10, v/v) to afford **15** (174.9 mg, 62%, white solid). HRMS (ESI^+^) calcd for C_85_H_122_NO_15_S (M^+^+H) 1428.8457, found 1428.8462. ^1^H NMR (500 MHz, CDCl_3_) δ 8.03–7.13 (m, 24H, C*H* aromatics), 6.61 (d, J = 9.4 Hz, 1H, -N*H*), 5.68–5.54 (m, 3H, H-2, H-3, H-9), 5.34 (td, *J* = 6.6, 3.1 Hz, 1H, H-10), 5.07 (d, *J* = 3.6 Hz, 1H, H-1), 4.63 (tt, *J* = 8.6, 3.7 Hz, 1H, H-8), 4.33 (s, 1H, H-5), 4.26 (m, *J* = 4.8 Hz, 1H, H-4), 4.17 (dq, *J* = 9.9, 4.8 Hz, 2H, H-6), 3.79 (dd, *J* = 10.9, 3.2 Hz, 1H, H-7^A^), 3.56 (dd, *J* = 10.9, 4.0 Hz, 1H, H-7^B^), 2.42 (s, 3H, C*H*_3_ of Ts), 2.24 (q, *J* = 7.0 Hz, 2H, -C*H*_2_-C_22_H_44_-CH_3_), 1.86 (q, *J* = 7.0 Hz, 2H, -C*H*_2_-C_12_H_24_-CH_3_), 1.35–1.16 (m, 68H, -CH_2_-C_12_ *H*_24_-CH_3_, -CH_2_-C_22_ *H*_44_-CH_3_), and 0.92–0.83 (m, 6H, -CH_2_-C_12_H_24_-C*H*_3_, -CH_2_-C_22_H_44_-C*H*_3_). ^13^C NMR (126 MHz, CDCl_3_) δ 133.19, 133.19, 132.94, 130.01, 129.77, 129.77, 129.77, 129.77, 129.52, 128.55, 128.30, 128.30, 128.06, 128.06, 128.06, 97.53, 73.85, 72.14, 70.18, 68.11, 67.99, 67.99, 67.50, 67.25, 48.21, 36.49, 29.40, 29.40, 28.43, 21.34, and 13.77.

[(1S,2S,3R)-1-[[[6-Azido-6-deoxy-2,3-bis-O-(Dibenzoyloxy)-β-D-galactopyranosyl]oxy]methyl].

*-2,3-bis(Dibenzoyloxy)heptadecyl]hexacosanamide (* ***4*** *).* Compound **15** (1 equiv, 108 mg) was dissolved in anhydrous DMF (4 ml), followed by the addition of NaN_3_ (3 equiv, 14.7 mg). The reaction mixture was stirred at 80°C overnight and cooled to room temperature. A measure of 40 ml of H_2_O was added, and two layers were separated. The aqueous layer was extracted with CH_2_Cl_2_. The organic extracts were combined and dried over anhydrous Na_2_SO_4_, and then concentrated *in vacuo*. The resulting residue was purified by column chromatography on silica gel hexanes/EtOAc (from 50/10 to 30/10, v/v) to afford **4** (85 mg, 86%). HRMS (ESI^+^) calcd for C_78_H_114_N_4_NaO_12_S (M^+^+Na) 1321.8433, found 1321.8439. ^1^H NMR (500 MHz, C_5_H_5_N) δ 8.24–7.26 (m, 20H, C*H* aromatics), 7.82 (d, J = 5.8 Hz, 1H, -N*H*), 6.44 (dd, *J* = 10.8, 3.9 Hz, 1H, H-2), 6.27 (d, *J* = 4.7 Hz, 1H, H-9), 6.16–6.05 (m, 2H, H-3, H-10), 5.73 (d, *J* = 3.8 Hz, 1H, H-1), 5.44 (d, *J* = 8.7 Hz, 1H, H-8), 4.81 (s, 1H, H-4), 4.60 (dd, *J* = 16.5, 6.4 Hz, 2H, H-5, H-7^A^), 4.15–3.99 (m, 2H, H-6^A^, H-7^B^), 3.62 (dd, *J* = 12.7, 4.6 Hz, 1H, H-6^B^), 2.50 (t, *J* = 7.3 Hz, 2H, -C*H*_2_-C_22_H_44_-CH_3_), 2.29 (td, *J* = 10.3, 5.5 Hz, 2H, -C*H*_2_-C_12_H_24_-CH_3_), 1.38–1.18 (m, 68H, -CH_2_-C_12_ *H*_24_-CH_3_, -CH_2_-C_22_ *H*_44_-CH_3_), and 0.95–0.75 (m, 6H, -CH_2_-C_12_H_24_-C*H*_3_, -CH_2_-C_22_H_44_-C*H*_3_). ^13^C NMR (126 MHz, C_5_H_5_N) δ 133.50, 133.25, 133.25, 129.84, 129.59, 128.86, 128.62, 128.62, 128.62, 128.37, 97.85, 73.92, 73.92, 72.21, 72.21, 70.74, 69.28, 67.93, 67.57, 51.57, 49.25, 36.31, 31.91, 29.47, 29.47, 28.74, 22.39, and 13.84.

*N-[(1S,2S,3R)-1-[[(6-Azido-6-deoxy-ɑ-D-galactopyranosyl)oxy]methyl]-2,3-dihydroxyheptadecyl]hexacosanamide (* ***1*** *).* Compound **4** (1 equiv, 118 mg) was dissolved in anhydrous MeOH/pyridine (1:1, 4 ml), 10 mg of solid sodium metal was added and dissolved in 1 ml of anhydrous methanol, and the resulting reaction mixture was stirred for 4 h at room temperature. Cationic resin was added to neutralize, was filtered, and then concentrated *in vacuo*. The resulting residue was purified by column chromatography on silica gel EtOAc/pyridine (from 100/1 to 90/1, v/v) to afford **1** (43.3 mg, 54%). HRMS (MALDI-TOF) calcd for C_50_H_98_N_4_NaO_8_ [M + Na]^+^ 905.7385, found 905.7391. ^1^H NMR (500 MHz, C_5_H_5_N) δ 8.57 (d, J = 8.8 Hz, 1H, -N*H*), 5.55 (d, *J* = 3.9 Hz, 1H, H-1), 5.31 (s, 1H, H-8), 4.73 (dd, *J* = 10.8, 5.5 Hz, 1H, H-7^A^), 4.61 (dd, *J* = 10.0, 4.0 Hz, 1H, H-2), 4.42–4.31 (m, 4H, H-3, H-5, H-7^A^, H-10), 4.26 (d, *J* = 3.3 Hz, 1H, H-4), 3.99 (dd, *J* = 12.7, 8.2 Hz, 1H, H-6^A^), 3.62 (dd, *J* = 12.6, 4.7 Hz, 1H, H-6^B^), 2.47 (d, *J* = 4.0 Hz, 2H, -C*H*_2_-C_22_H_44_-CH_3_), 1.68–1.63 (m, 2H, -C*H*_2_-C_12_H_24_-CH_3_), 1.35–1.14 (m, 68H, -CH_2_-C_12_ *H*_24_-CH_3_, -CH_2_-C_22_ *H*_44_-CH_3_), and 0.86 (dt, *J* = 17.2, 6.9 Hz, 6H, -CH_2_-C_12_H_24_-C*H*_3_, -CH_2_-C_22_H_44_-C*H*_3_). ^13^C NMR (126 MHz, C_5_H_5_N) δ 100.95, 76.29, 72.14, 70.67, 70.43, 69.45, 68.47, 68.11, 51.87, 51.38, 50.65, 36.24, 31.85, 30.38, 29.40, 29.40, 22.32, 18.90, and 13.53.

*N-[(1S,2S,3R)-1-[[(6-Amino-6-deoxy-ɑ-D-galactopyranosyl)oxy]methyl]-2,3-dihydroxyheptadecyl]hexacosanamide (* ***2*** *).* Compound **1** (1 equiv, 26 mg) was dissolved in CH_2_Cl_2_/MeOH (1:1, 2 ml) and was stirred with a tablespoon of Pd/C under H_2_ atmosphere at room temperature for 5 h. After the disappearance of starting material monitored by TLC, the reaction mixture was filtered through a 0.22-um nylon microporous membrane. The mixture was concentrated under reduced pressure, and the residue was purified by silica gel column chromatography using a gradient of hexanes/EtOAc (from 50/10 to 10/10, v/v, added 2% TEA) to afford **2** (21.9 mg, 87%). HRMS (ESI^+^) calcd for C_50_H_101_N_2_O_8_ (M^+^+H) 857.7480, found 857.7481. ^1^H NMR (500 MHz, C_5_H_5_N) δ 8.56 (d, J = 8.5 Hz, 1H, -N*H*), 5.27 (d, *J* = 5.0 Hz, 2H, H-1, H-4), 5.18 (s, 1H, H-8), 4.84 (d, *J* = 5.4 Hz, 1H, H-5), 4.76 (dd, *J* = 10.4, 4.2 Hz, 2H, H-6), 4.59 (s, 1H, H-2), 4.43–4.34 (m, 3H, H-3, H-7^A^, H-9), 4.25 (d, *J* = 8.9 Hz, 1H, H-10), 4.19 (d, *J* = 9.4 Hz, 1H, H-7^B^), 2.40 (t, *J* = 7.4 Hz, 2H, -C*H*_2_-C_22_H_44_-CH_3_), 1.91 (d, *J* = 9.7 Hz, 2H, -C*H*_2_-C_12_H_24_-CH_3_), 1.36–1.14 (m, 68H, -CH_2_-C_12_ *H*_24_-CH_3_, -CH_2_-C_22_ *H*_44_-CH_3_), and 0.87 (d, *J* = 7.5 Hz, 6H, -CH_2_-C_12_H_24_-C*H*_3_, -CH_2_-C_22_H_44_-C*H*_*3*_). ^13^C NMR (126 MHz, C_5_H_5_N) δ 98.10, 82.64, 78.25, 75.96, 72.45, 70.78, 69.64, 69.29, 51.89, 36.61, 31.87, 29.76, 29.58, 26.24, 22.55, and 13.95.

*[(1S,2S,3R)-1-[[(6-(Pent-4-ynamidomethyl)-6-deoxy-ɑ-Dgalactopyranosyl)oxy]methyl]-2,3 dihydroxyheptadecyl]hexacosanamide (* ***3*** *).* Compound **2** (1 equiv, 12.6 mg) was dissolved in anhydrous DMF (2 ml) and was stirred with 4-pentynoic acid (5 equiv, 14.3 mg) after NHS activation. HATU (1.5 equiv, 8.38 mg) and TEA (10 equiv, 20.5 ul) were added, stirring the reaction overnight at room temperature. Then, it was quenched with methanol and concentrated *in vacuo*, and the residue was purified by SePhadexLH-20 using CH_2_Cl_2_/methanol (1:2, v/v) to afford **3** (12.8 mg, 93%). HRMS (ESI^+^) calcd for C_55_H_104_N_2_NaO_9_ (M^+^+Na) 959.7742, found 959.7746. ^1^H NMR (500 MHz, C_5_H_5_N) δ 9.06 (s, 1H, -N*H*), 8.61 (d, J = 8.5 Hz, 1H, -N*H*), 5.51 (d, *J* = 4.1 Hz, 1H, H-1), 5.25 (s, 1H, H-8), 4.62 (dd, *J* = 16.2, 9.4 Hz, 2H, H-2, H-7^A^), 4.50 (t, *J* = 6.5 Hz, 1H, H-5), 4.32 (d, *J* = 14.2 Hz, 5H, H-3, H-4, H-7^B^, H-9, H-10), 4.28–4.15 (m, 1H, H-6^A^), 3.94 (d, *J* = 6.7 Hz, 1H, H-6^B^), 3.00–2.55 (m, 4H, H-11, H-12), 2.48 (q, *J* = 7.9 Hz, 2H, -C*H*_2_-C_22_H_44_-CH_3_), 1.93 (s, 2H, -C*H*_2_-C_12_H_24_-CH_3_), 1.88–1.04 (m, 68H, -CH_2_-C_12_ *H*_24_-CH_3_, -CH_2_-C_22_ *H*_44_-CH_3_), and 0.88 (t, *J* = 6.8 Hz, 9H, -CH_2_-C_12_H_24_-C*H*_3_, -CH_2_-C_22_H_44_-C*H*_*3*_). ^13^C NMR (126 MHz, C_5_H_5_N) δ 101.05, 76.45, 72.23, 70.83, 70.30, 69.77, 68.02, 50.97, 41.14, 40.87, 36.39, 35.16, 33.93, 32.00, 30.42, 29.72, 29.54, 26.03, 22.51, 14.78, and 13.90.

### 2.2 Pharmacology

#### 2.2.1 *In vivo* experiment

Bald/C mice were purchased from Sibeifu Co., LTD. The mice were kept under specific pathogen-free conditions and used at 7 weeks of age. KRN7000 and synthesized glycolipidases (1.0 mg) were dissolved in dimethyl sulfoxide (DMSO, 1.0 ml) at 80°C. After 30 min at 80°C, the solutions were cooled to room temperature and diluted to 200 μg/ml with phosphate-buffered saline (PBS) containing 0.5% Tween 20 (polymethylene sorbitan monolaurate). The obtained solutions were diluted to 10 μg/ml with PBS just before injecting into the mice. Each glycolipid solution (10 μg/ml, 200 μL) was administered intravenously. Peripheral blood was collected from the retro-orbital plexus of mice at 3, 6, 12, 24, 36, 48, and 60 h using capillary tubes, and plasma was prepared. The animal protocol involved in this research was approved (HNUAUCC-2021–00048).

#### 2.2.2 Cytokine measurement

The cytokine concentrations in plasma were measured by using the mouse ELISA kit (Dekewe company) for IFN-γ and IL-4.

## 3 Results and discussion

**SCHEME 1:**
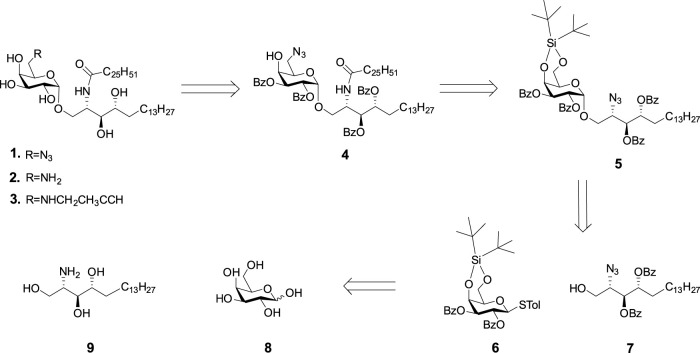
Strategy for the preparation of KRN7000 and its 6'-modified analogues.

The retro-synthetic analysis of C-6-modified KRN7000 (**1**, **2**, and **3**) is shown in Scheme 1. Briefly, the azide, amine, or alkyne handles can be introduced at the late stage of synthesis. Fully protected compound **5** can be prepared by the glycosylation of donor **6** and acceptor **7**. The glycosyl donor **6** will be protected by 4,6-DTBS and 2,3-Bz groups. The azide was selected as a temporary lipid amine protecting group for acceptor **7**, which can be selectively reduced to an amine and coupled with ceric acid. The two secondary hydroxyl groups were protected by Bz groups, which can be removed by the base at the final stage.

**SCHEME 2:**
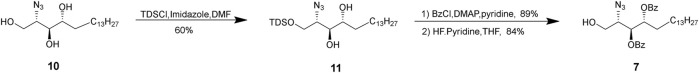
Synthesis of acceptor **7**.

The synthesis of acceptor **7** is shown in Scheme 2 **.** The amine of commercially available phytosphingosine was first transformed to azide, following the literature report (Worth et al., 2019). Then, the primary hydroxyl group was selectively protected by a thexyldimethylsilyl (TDS) group to give **9**. Other groups have also employed a bulkier group such as the *tert*-butyldiphenylsilyl (TBDPS) group, which was found rather sluggish in our hands. Then, the other two secondary hydroxyl groups were protected by Bz groups, followed by TDS removal by HF in pyridine to give glycosyl acceptor **7**.

**SCHEME 3:**
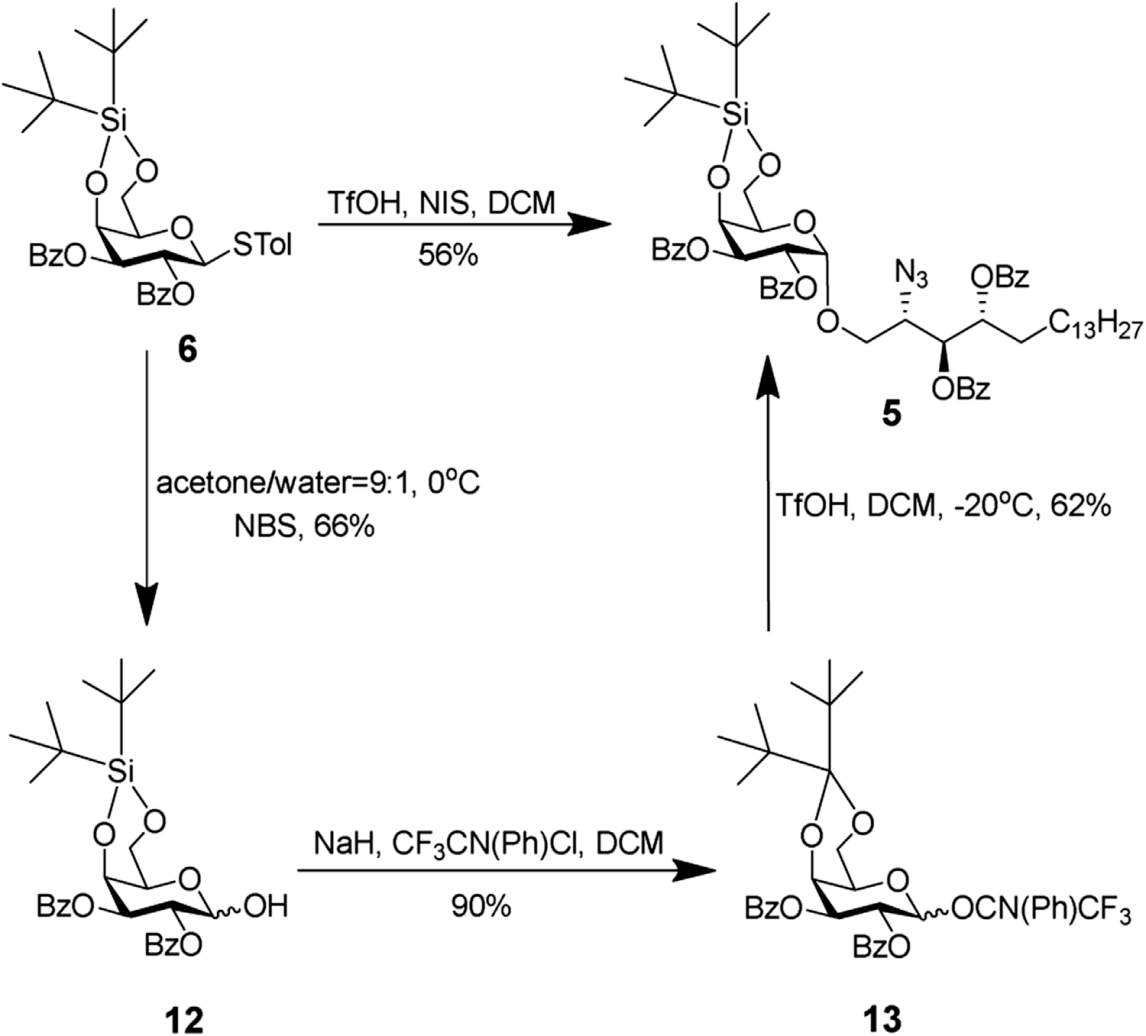
Glycosylation of donor **6** or N-phenyl with the acceptor.

Donor **6** was prepared according to previously reported procedures (Veerapen et al., 2009). The glycosylation strategy is shown in Scheme 3. The reaction between donor **6** and acceptor **7** turned out to be quite challenging. The reaction could not come to completion even with additional NIS (4 equiv) and TfOH (1 equiv). The best yield by this approach was 56%. Next, we explored whether the imidate donor could be a better option. Various methods to hydrolyze the thiolacetals were conducted by using N-bromosuccinimide (NBS) or 1,3-di-bromo-5,5-dimethylhydantoin (DBDMH). The yield was low due to undesired removal of DTBS. We also explored N-iodosuccinimide (NIS) and trifluoroacetic acid (TFA) in dichloromethane (DCM) (Dinkelaar et al., 2008). It should be noted that the NIS/TFA-mediated hydrolysis proceeded through the formation of the rather stable anomeric α-trifluoroacetate, which was hydrolyzed by triethylamine to afford the desired product (Gold et al., 2011). Unfortunately, no reaction was observed under this condition indicating inert reactivity of donor **6**, due to the electron withdrawing effect of C-2,3 Bz protecting groups. With the hemi-acetyl in hand, the N-phenyl donor was obtained under a standard condition.

Glycosylation between the N-phenyl donor and acceptor **7** gave a moderate yield of 62%. Both glycosylations afforded the same compound **5**, the NMR data on which are shown in Supplementary Figure S4. Comparing the total yield and labor involved, we decided to proceed with donor **6** for further glycosylation.

Based on TLC and NMR analysis, no β product was observed. Only ɑ product (^3^ *J*_H-1,H-2_ = 3.6 Hz) was generated even with the C-2 Bz protecting group, indicating the bulky 4,6-DTBS prevented the acceptor’s attack from the β-face to dominate the selectivity. Then, the azide of compound **5** was reduced to amine to couple with ceric acid to give compound **10**. Various methods were explored including Staudinger reduction or NiCl_2_/NaBH_4_-mediated reduction, which all gave low yield. Finally, we employed hydrogenation by using palladium on carbon as the catalyst. It should be noted that the product was not stable for prolonged storage, probably due to Bz migration from adjacent esters to the free amine. For this reason, we added 1.2 equiv 1M hydrochloride solution during the reaction to protonate the free amine. The product was stable thereafter. The coupling between the amine with ceric acid was performed in standard coupling conditions by using EDC/HOBT in a mixture of THF and DMF, followed by the removal of DTBS.

**SCHEME 4:**
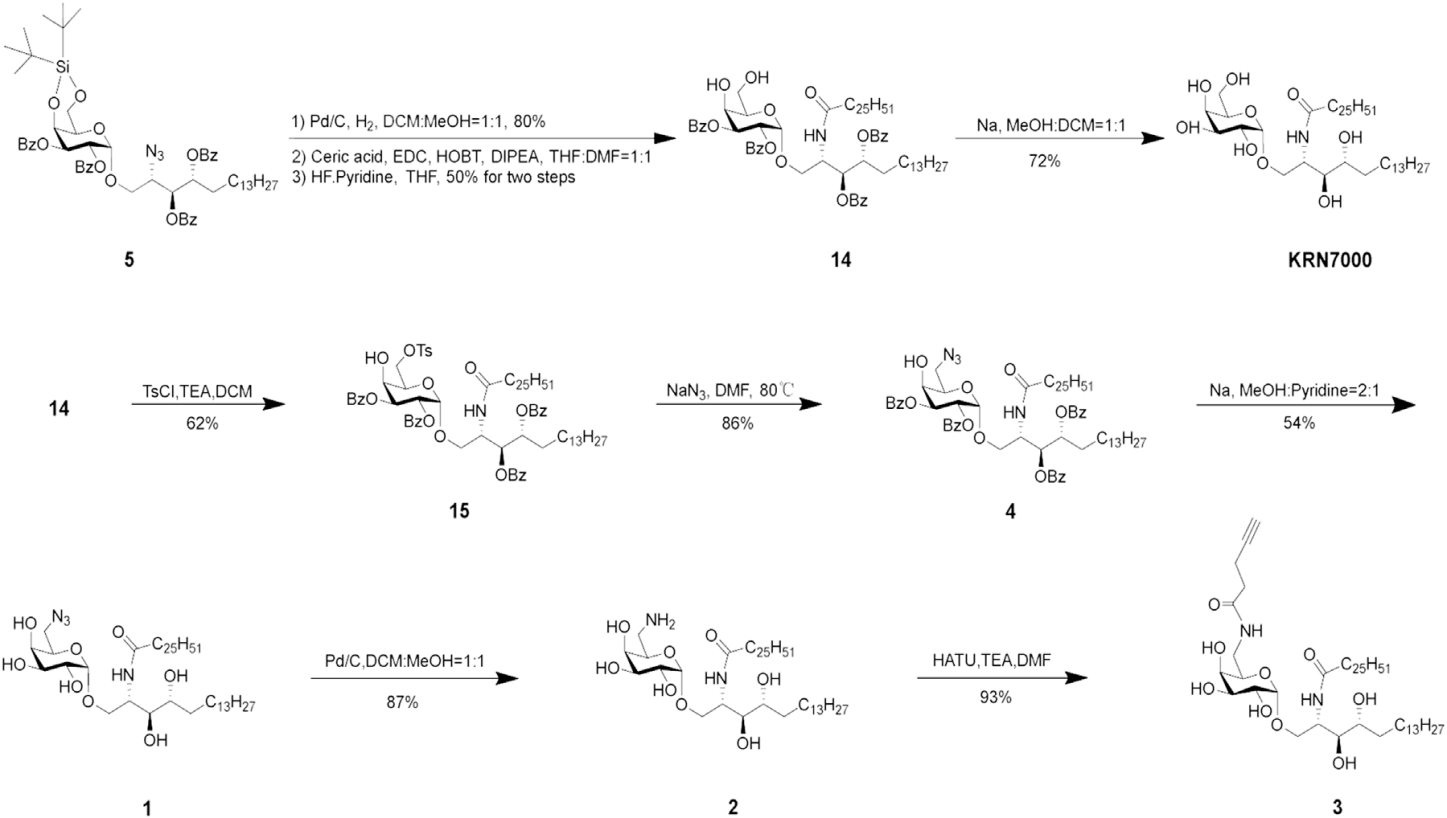
Synthesis of target compounds **1**, **2**, and **3**.

The synthesis of target compounds **1**, **2,** and **3** is shown in Scheme 4. KRN7000 can be obtained by removing all Bz protecting groups. For the C-6-modified KRN7000 analog **1**, the 4,6-DTBS group was selectively and smoothly removed by using HF in pyridine, followed by the C-6 selective tosylation, which can be replaced by the azide group under elevated temperature (80°C). The azide can also be transformed to other unsaturated functional groups at this stage, by first reducing the azide to amine and then coupling with appropriate groups (not performed). All Bz groups can be removed to give compound **1** by using Na in methanol and pyridine.

The azide group of compound **1** can also be further reduced to an amine to give **2**, which can be coupled with an alkyne to give **3**.

**FIGURE 1:**
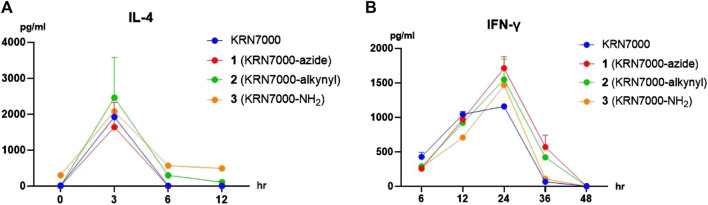
Cytokine production after injection of synthetic analogs in mice *in vivo* (2 μg/mouse, iv). Serum concentrations of IL-4 **(A)** and IFN-γ **(B)** were measured by ELISA at the indicated time points. Data are means ± standard deviation (SD) from three mice.

Various reports have demonstrated the C-6 modifications do not impact α-galactosylceramide derivatives’ biological activity. With the analogs in hand, we decided to investigate their ability to induce cytokine production by mouse lymphocytes *in vivo*. The cytokine concentration in Balb/C mice sera was monitored after intravenous (iv) administration of KRN7000 or synthesized analogs in phosphate-buffered saline (PBS) solutions (Tashiro et al., 2012). Intravenous injection of KRN7000 results in systemic iNKT cell activation. They can rapidly but transiently produce cytokines, with an initial burst of IL-4 (1–8 h), followed by IFN- γ (12–36 h activation) (Van Kaer and Wu, 2018). The sera samples were collected at 3, 6, 12, 24, 36, 48, and 60 h. The cytokine concentration was determined by ELISA. As can be seen from Figure 1, all three compounds demonstrated similar biological activity, indicating the success of the strategy.

## 4 Conclusion

α-Galactosylceramide is an effective iNKT cell by forming a ternary complex with CD1d and T-cell receptor. Multiple vaccine clinical trials were under investigation by using α-galactosylceramide as an adjuvant, which indicated it might be not potent enough to induce effective vaccine protection. To solve this issue, many research groups are attempting the covalent conjugation of an antigen and α-galactosylceramide. According to the literature, the C6 modifications of α-galactosylceramide do not impact the biological activity. However, the efficient synthesis of KRN7000 analogs remains an obstacle due to the synthetic challenges, especially the difficult α-glycosidic bond. Bn has been commonly used, and azide is used as the C-6 protecting groups to synthesize the compounds. After the final hydrogenation to remove benzyl groups, the C6 azide will be simultaneously reduced to amine for further modification. However, we found that KRN7000 has a poor solubility in common solvents like dichloromethane, methanol, or ethyl acetate, making the modification or purification difficult. Alternatively, the C6 modifications can be performed prior to hydrogenation. By using the benzyl protecting group, the final hydrogenation is not compatible with un-saturated moieties, such as the commonly used clickable groups like the azide or alkyne.

Here, we report an efficient procedure for the synthesis of KRN7000 and its analogs based on the 4,6-DTBS, 2,3-Bz protected donor for α-selectivity. This procedure generated only the α-product and is compatible with the C-6 azide modification. All compounds demonstrated similar biological activity. The strategy laid foundation for the synthesis of antigen and KRN7000 conjugates.

## Data Availability

The original contributions presented in the study are included in the article/Supplementary Material; further inquiries can be directed to the corresponding author.
