# Supplementary material for: Efficient synthesis of α-galactosylceramide and its C-6 modified analogs
Source: Front Chem. 2022 Nov 25;10:1039731. doi: 10.3389/fchem.2022.1039731 (PMC9732566; doi:10.3389/fchem.2022.1039731)
Supplement: Supplementary file 1 [file DataSheet1.docx]

Supplementary Material

# ^1^H NMR and HSQC


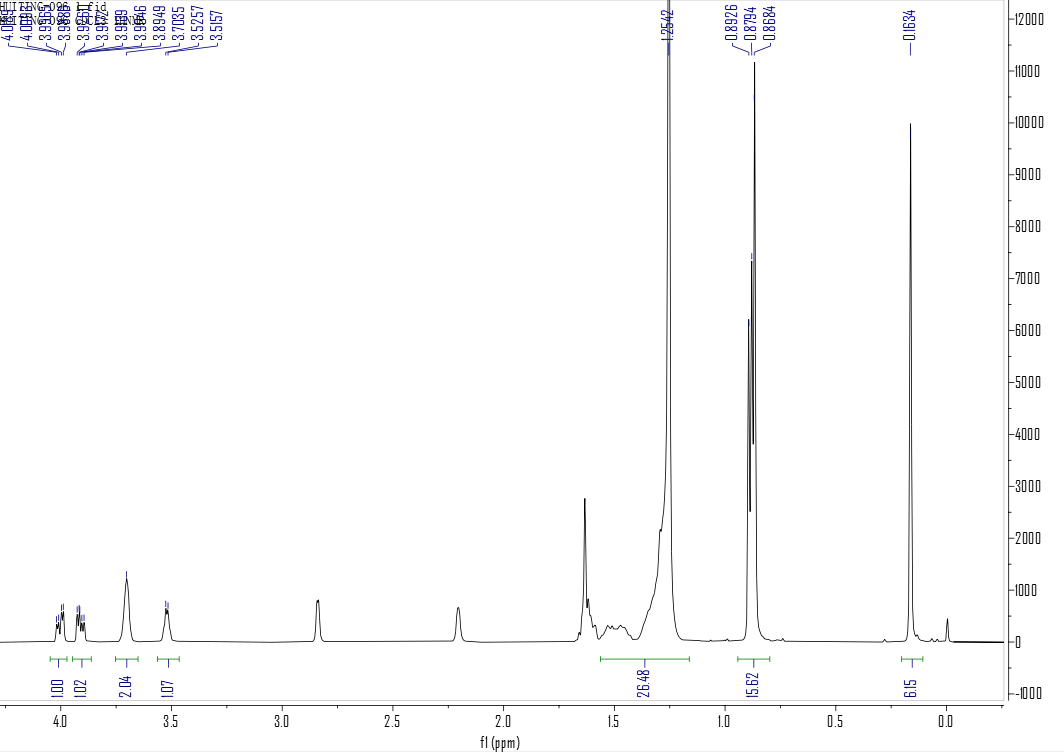

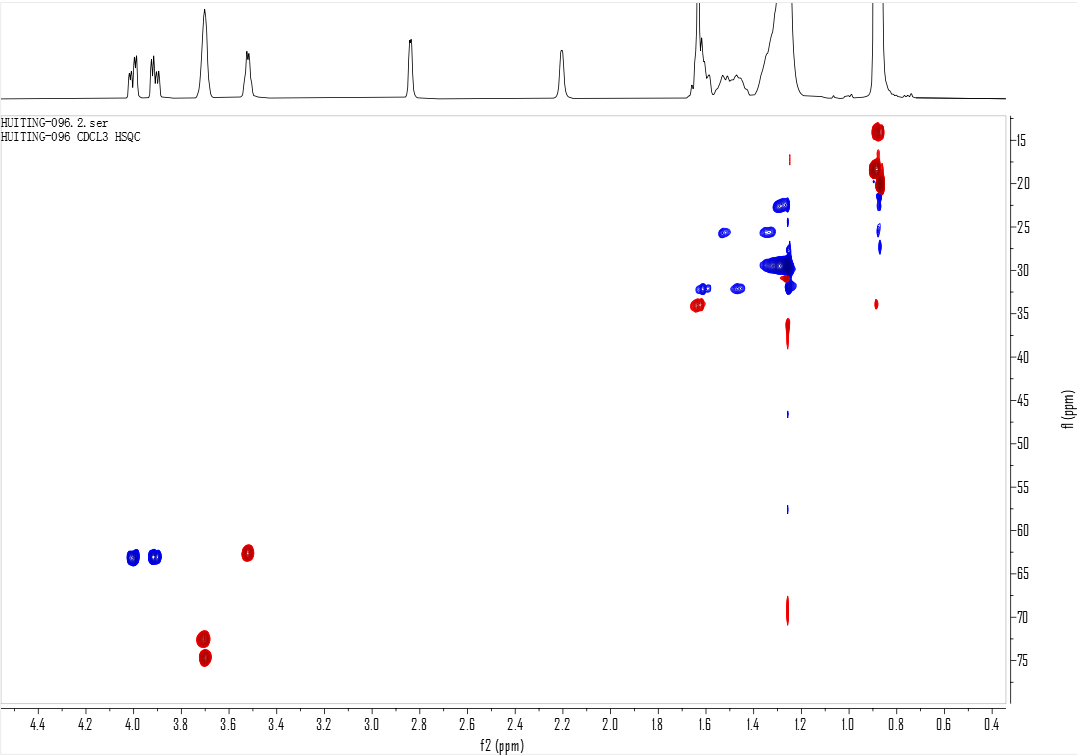


**
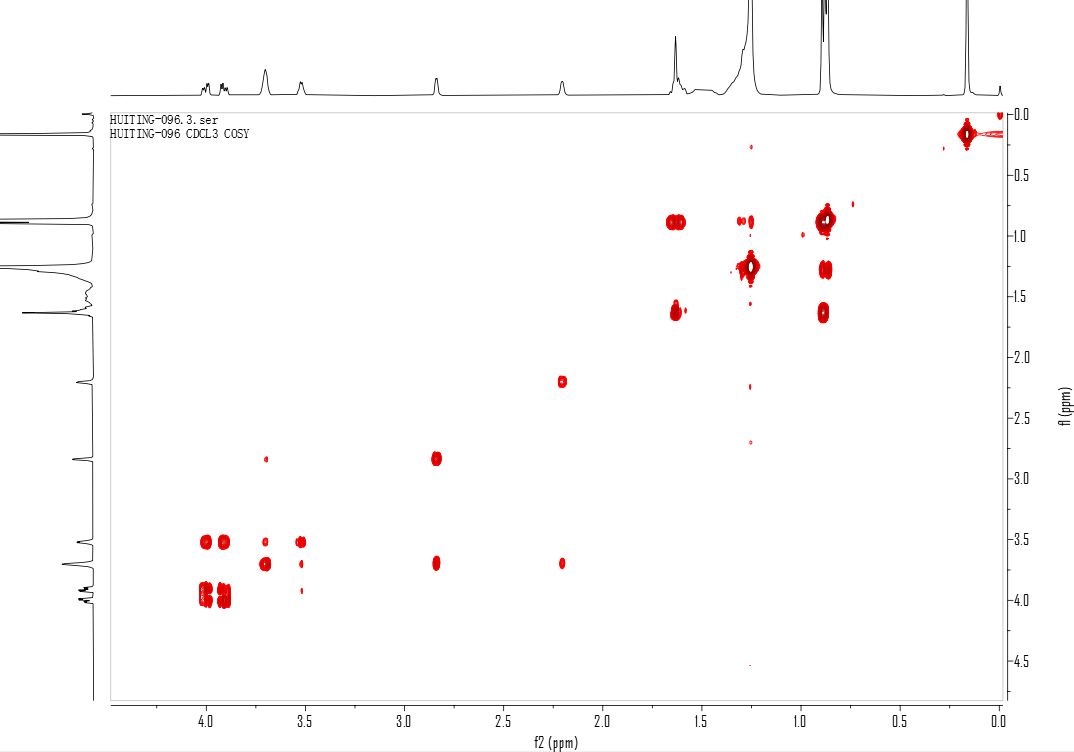
**

**Figure S1.** NMR spectrum of compound (**11**)


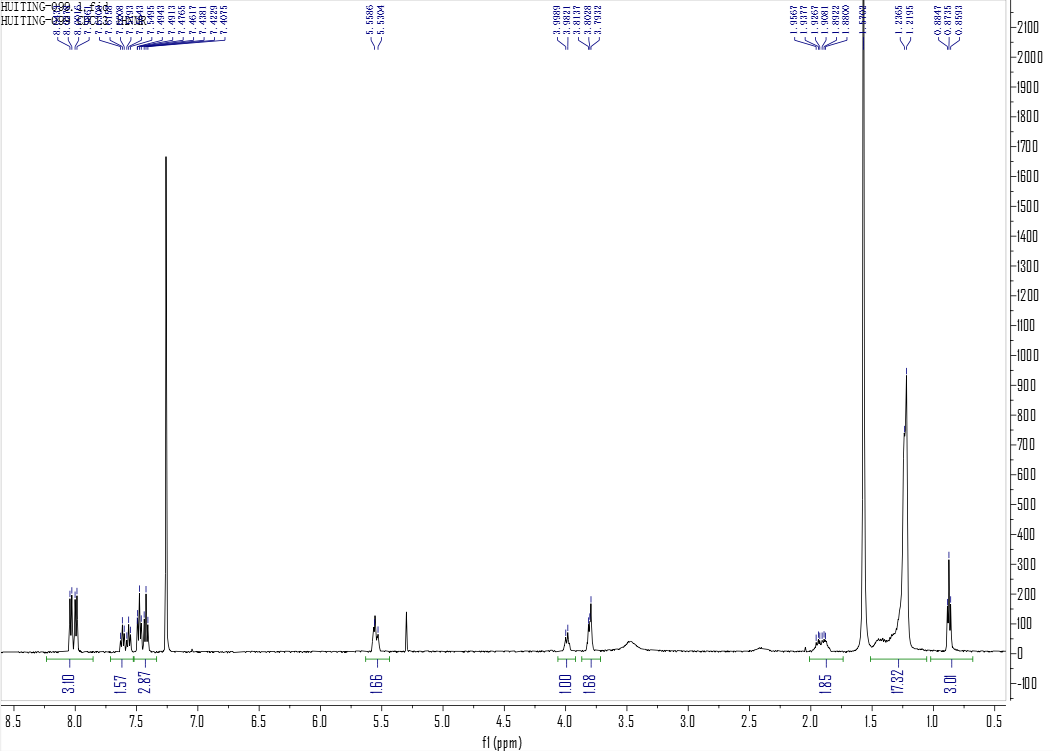


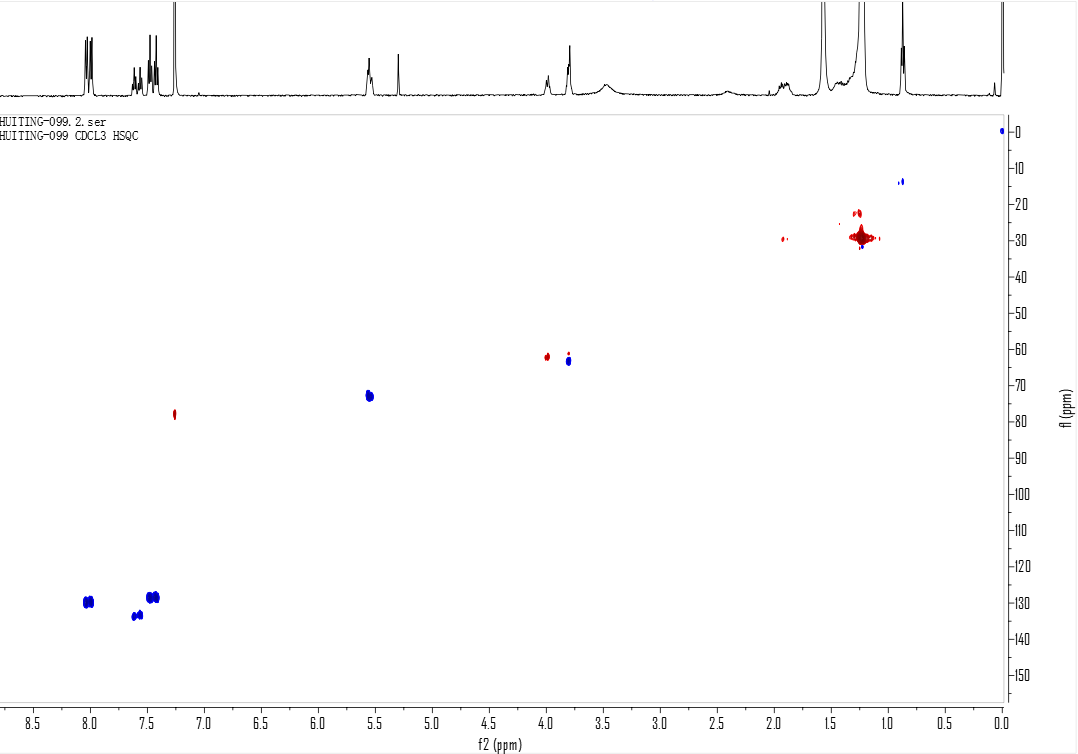


**
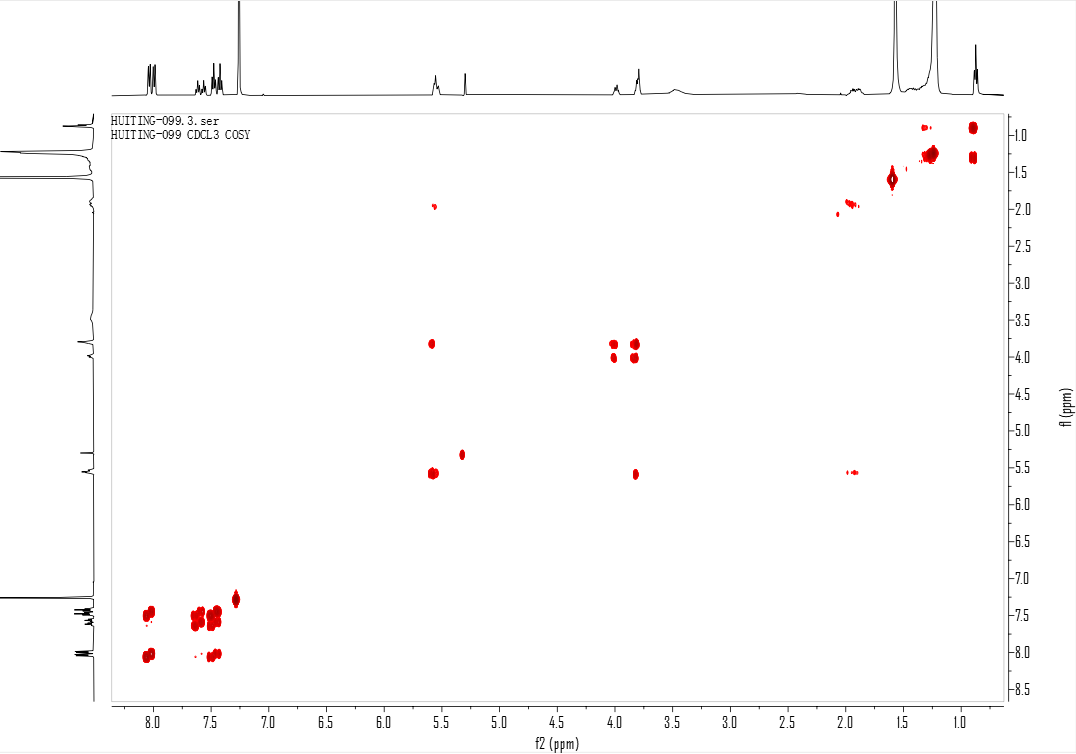
**

**Figure S2.** NMR spectrum of compound (**7**)


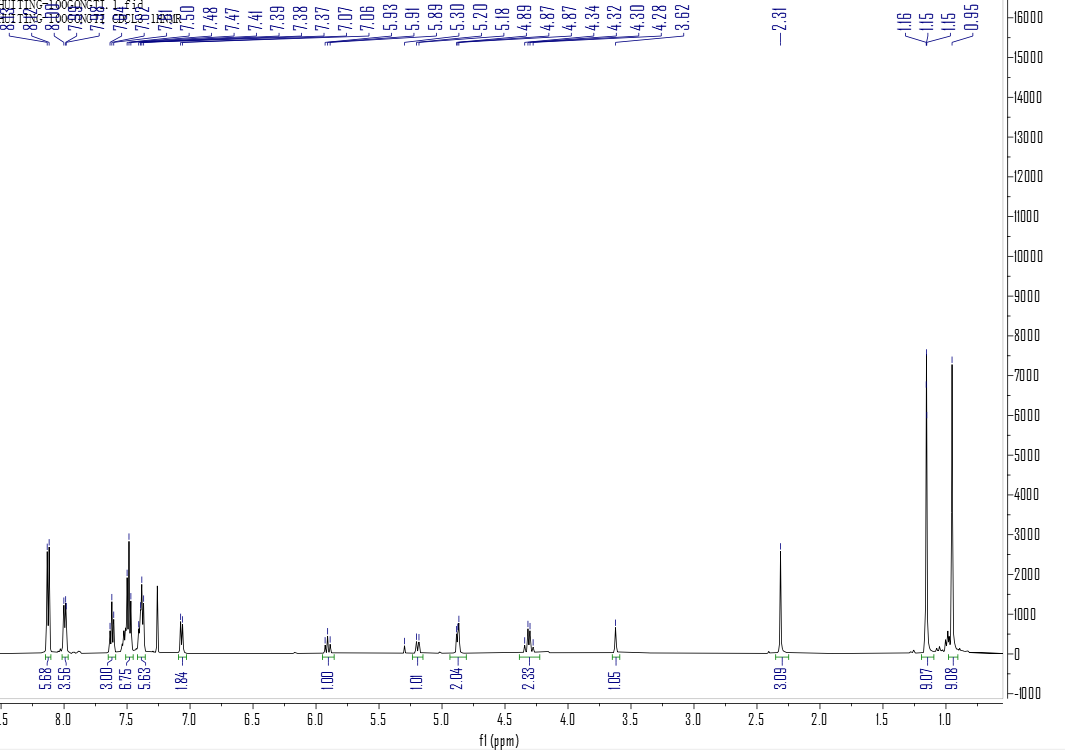


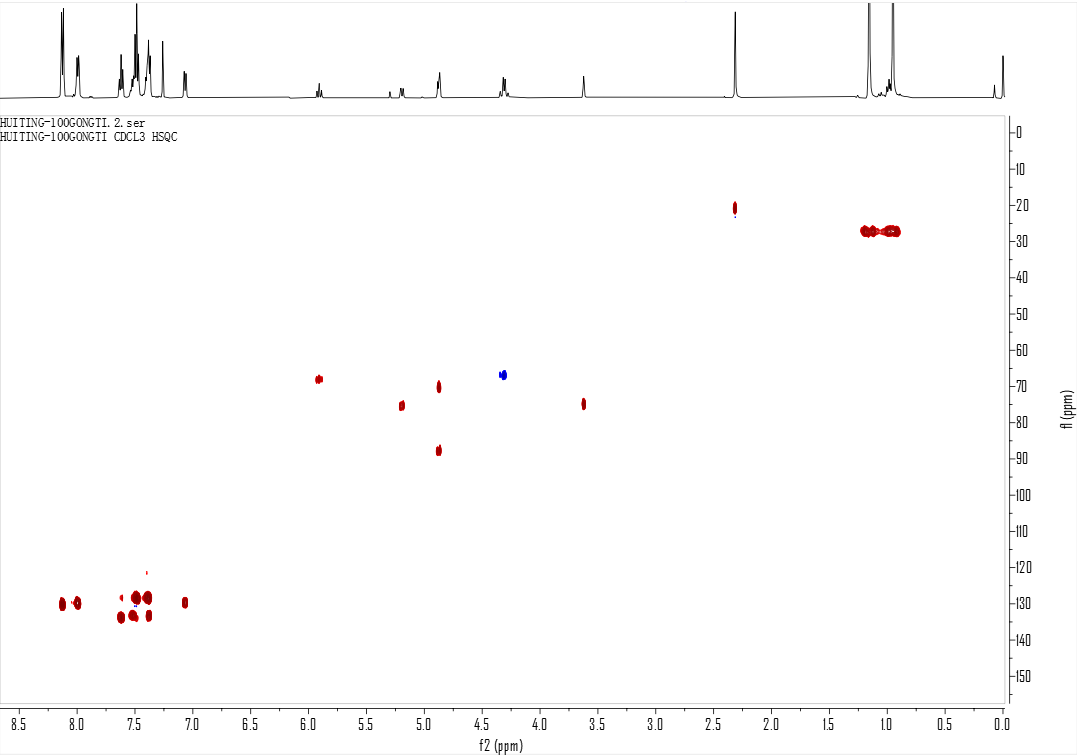


**
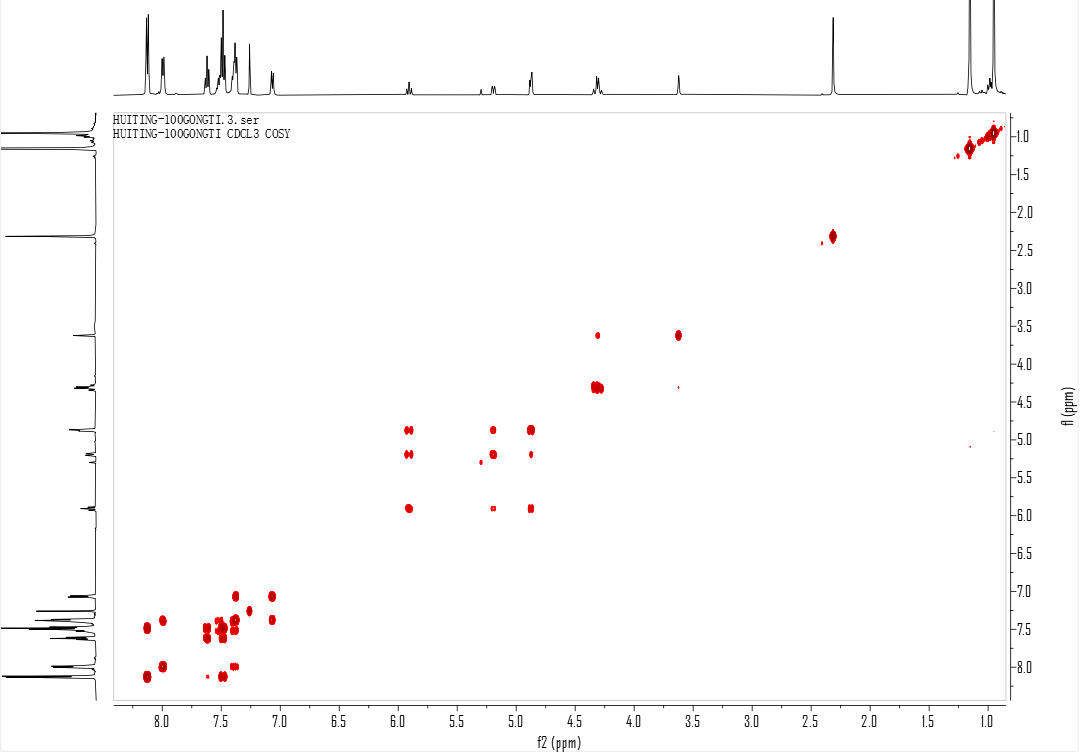
**

**Figure S3.** NMR spectrum of compound (**6**)


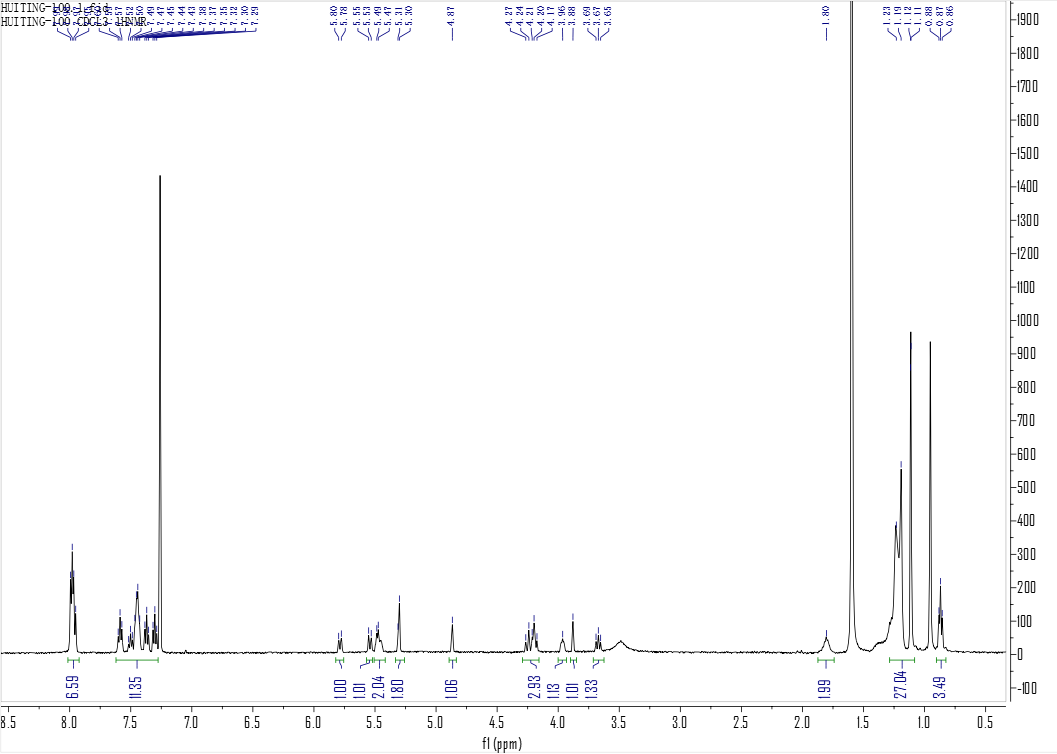


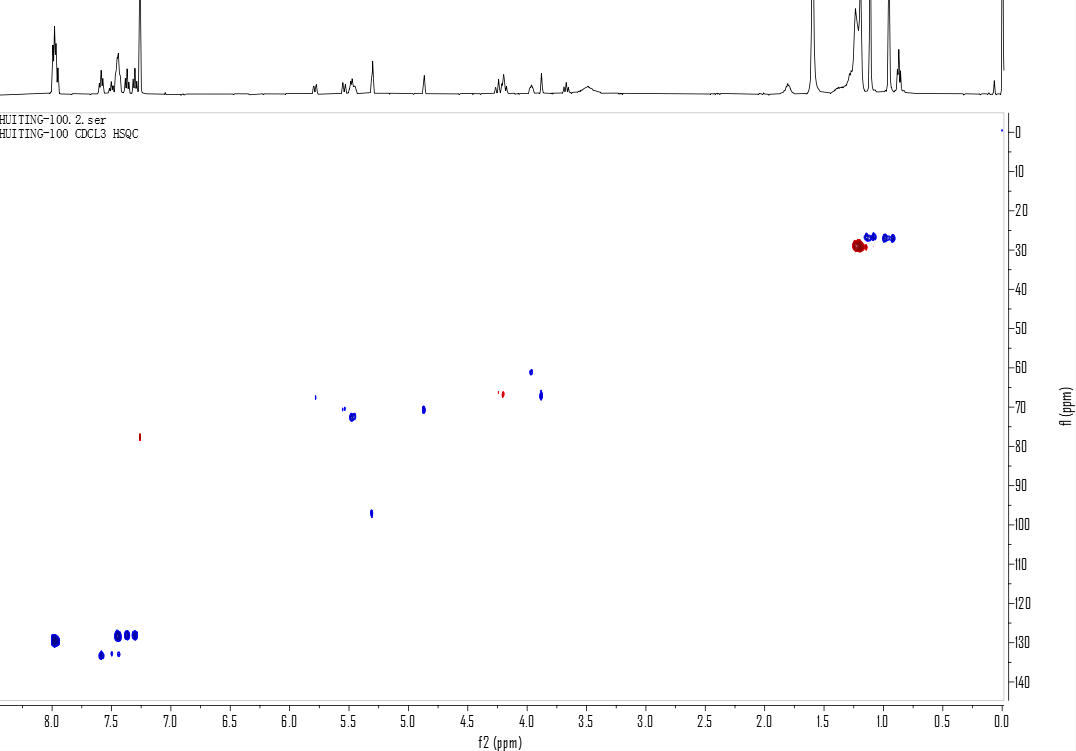


**
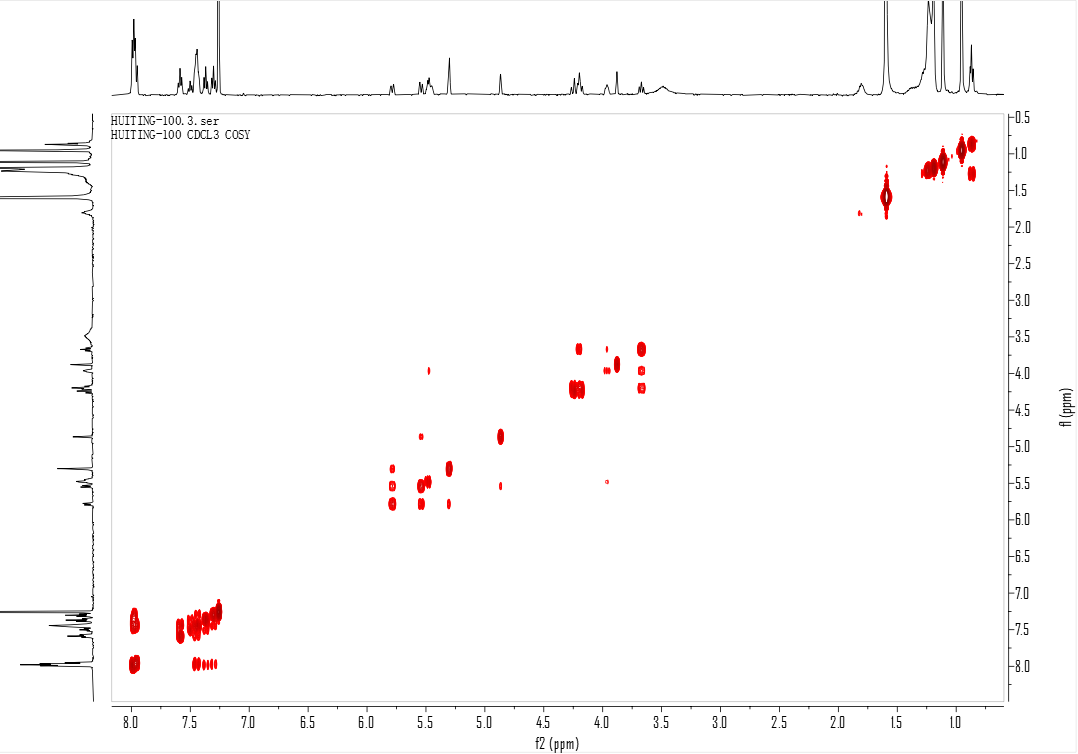
**

**Figure S4.** NMR spectrum of compound (**5**)


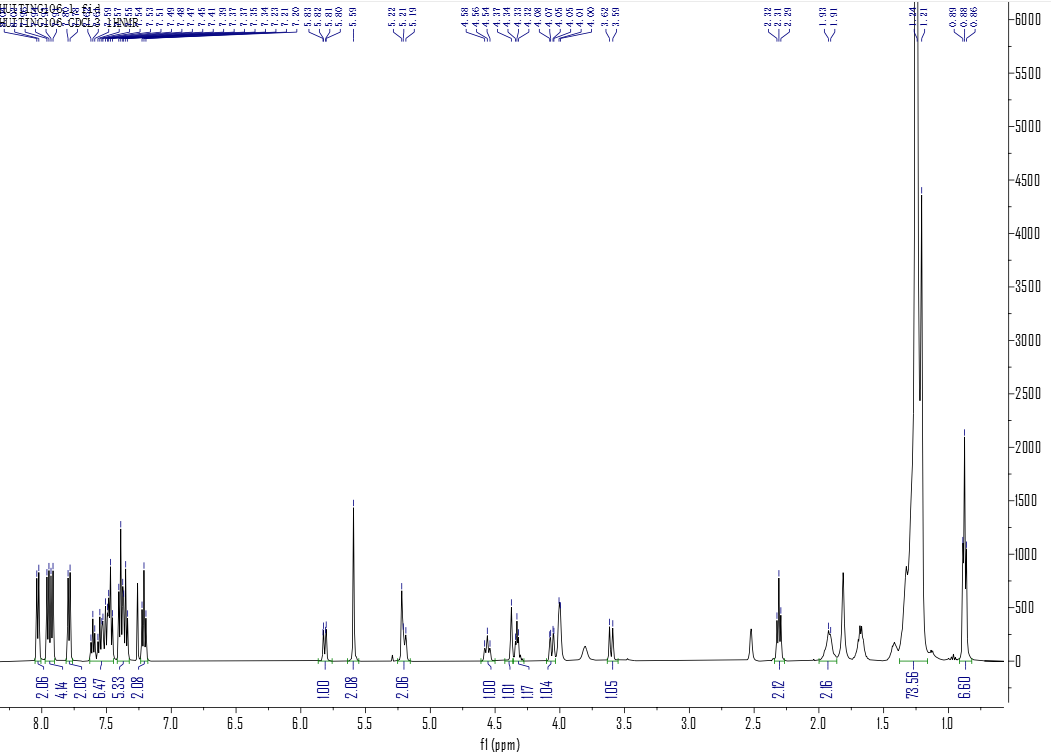


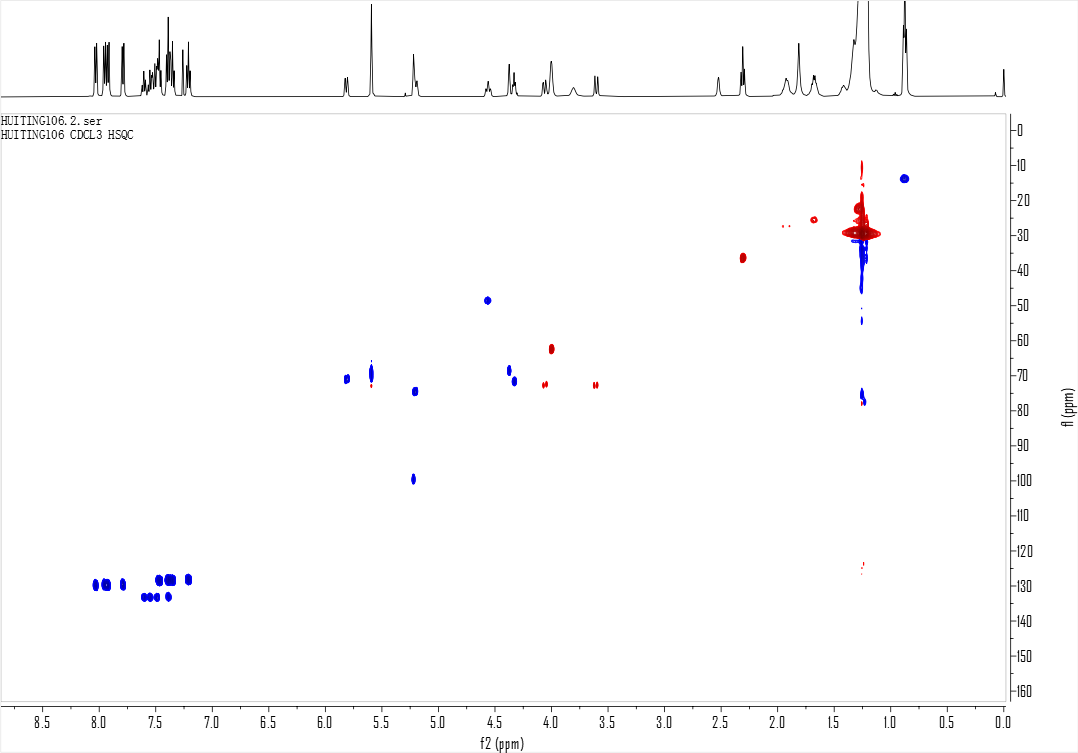


**
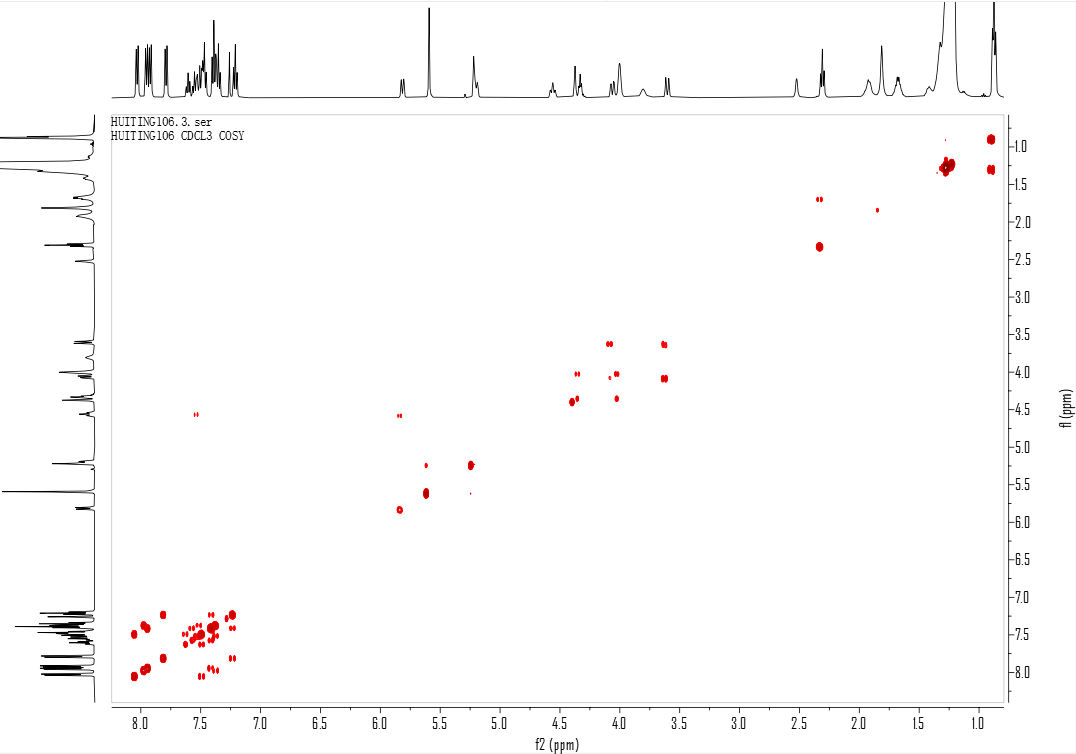
**

**Figure S5.** NMR spectrum of compound (**14**)


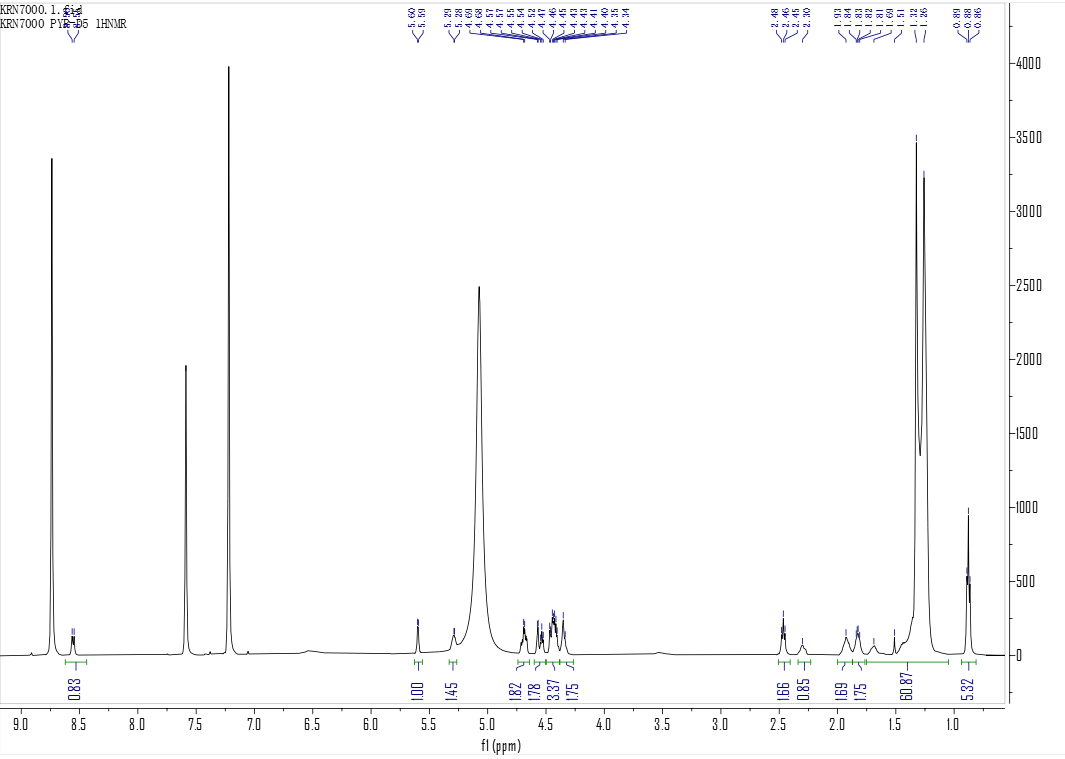


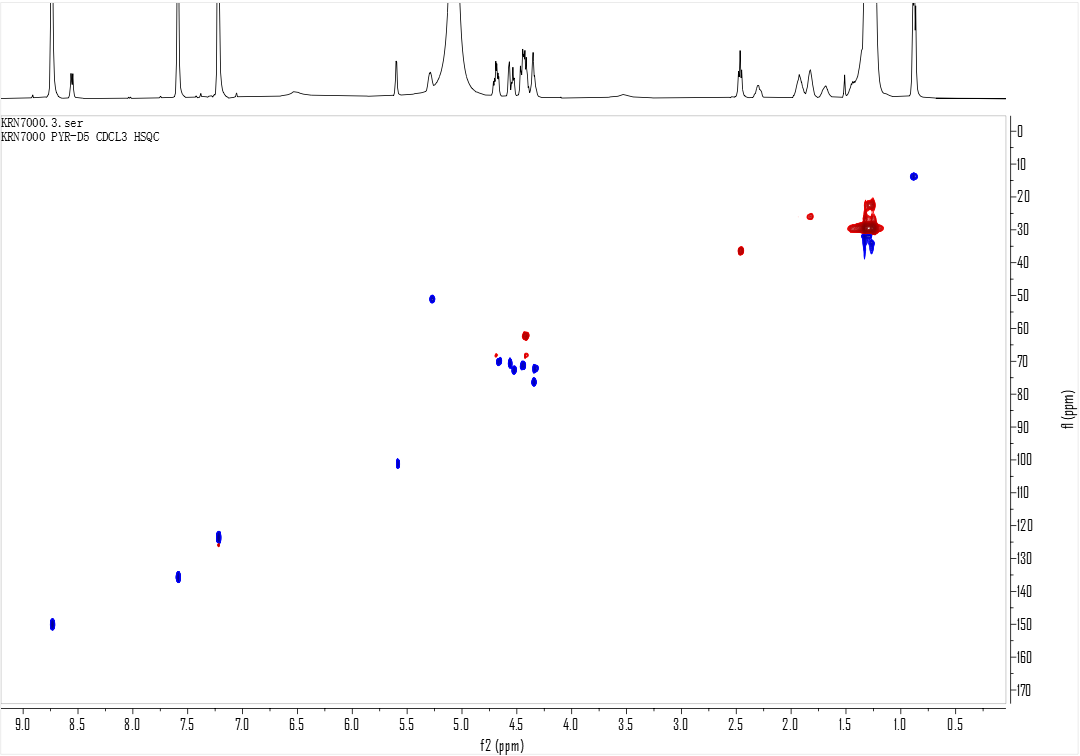


**
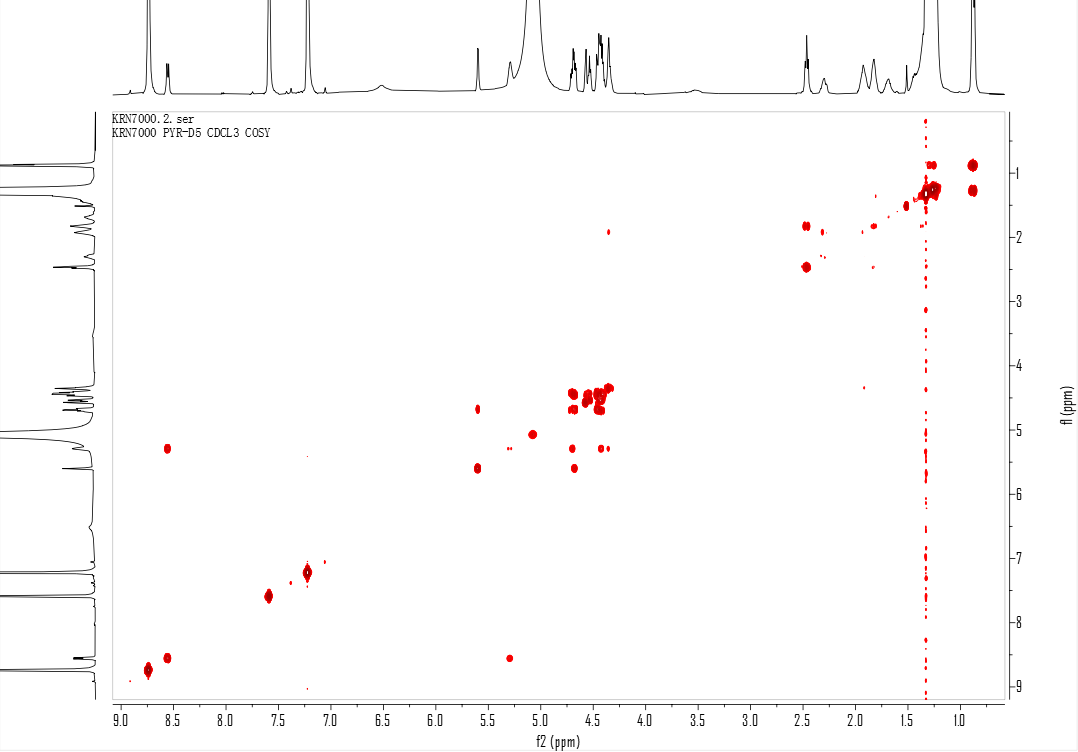
**

**Figure S6.** NMR spectrum of compound (**KRN7000**)


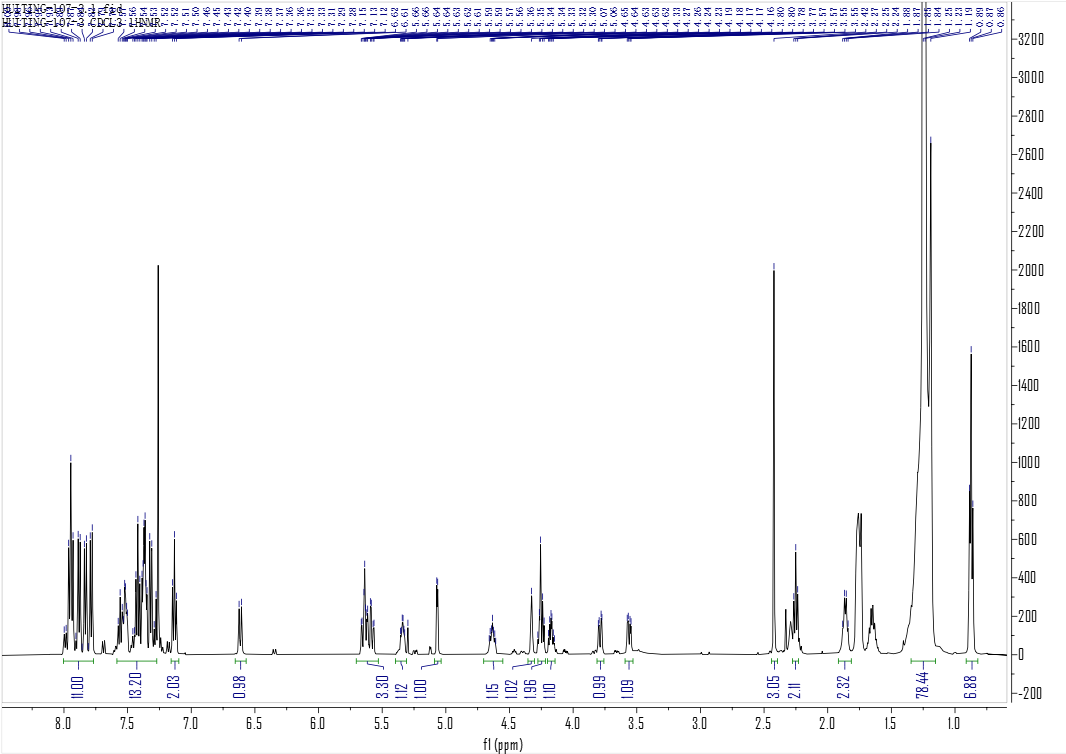


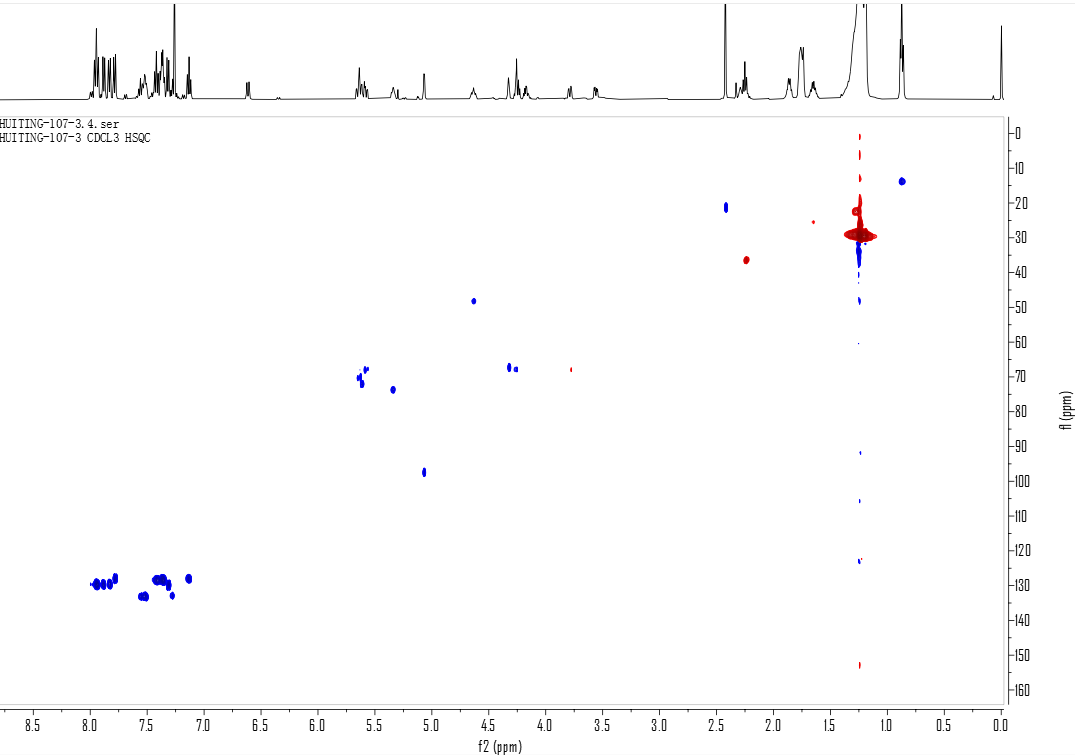


**
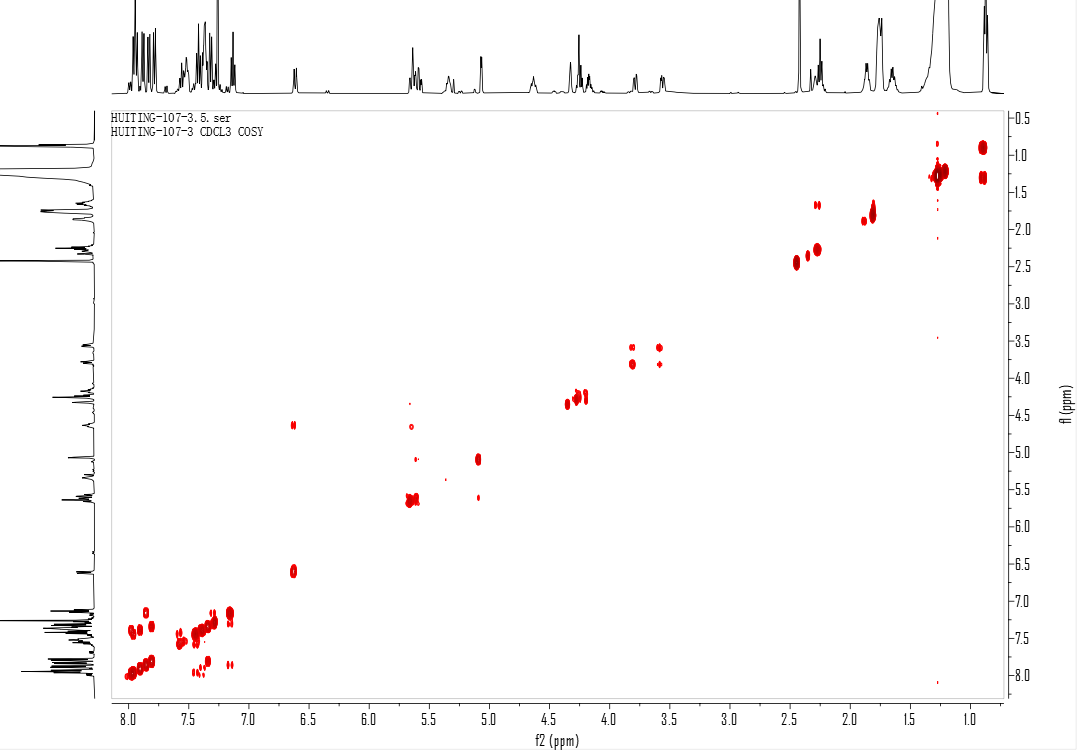
**

**Figure S7.** NMR spectrum of compound (**15**)


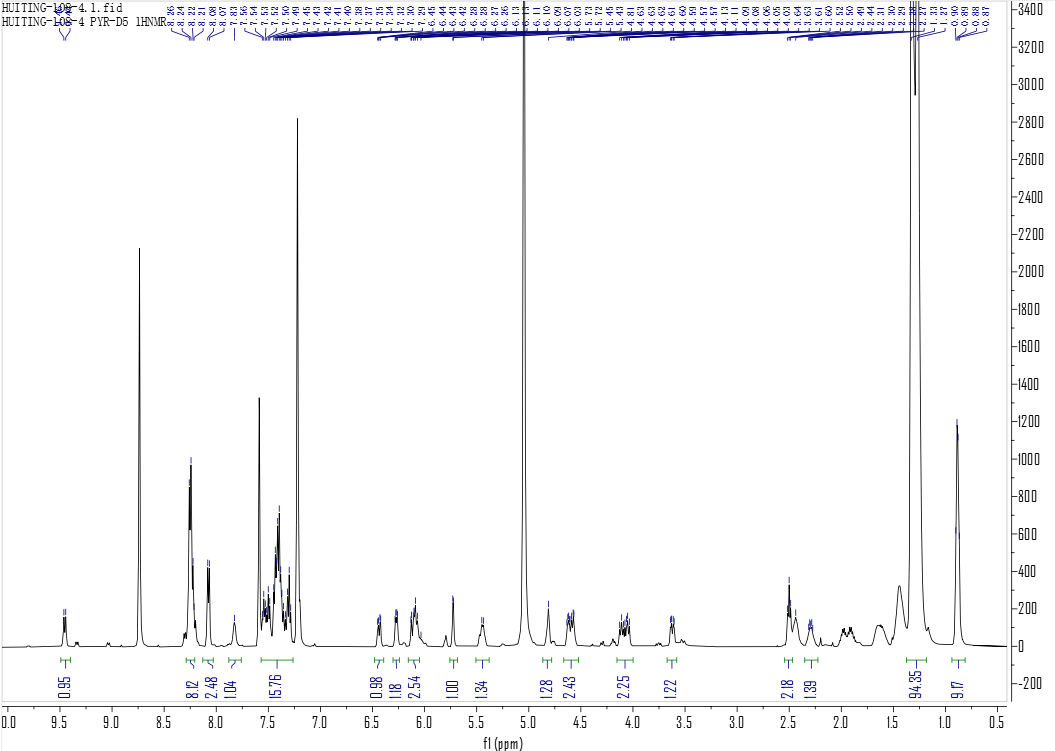


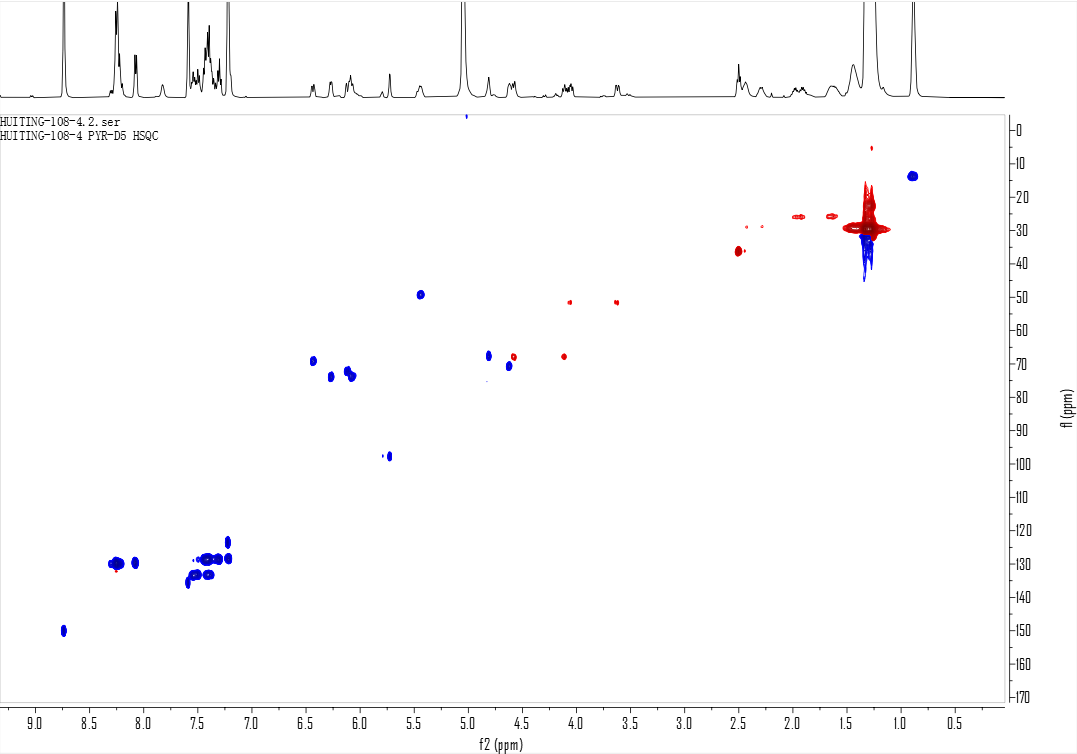


**
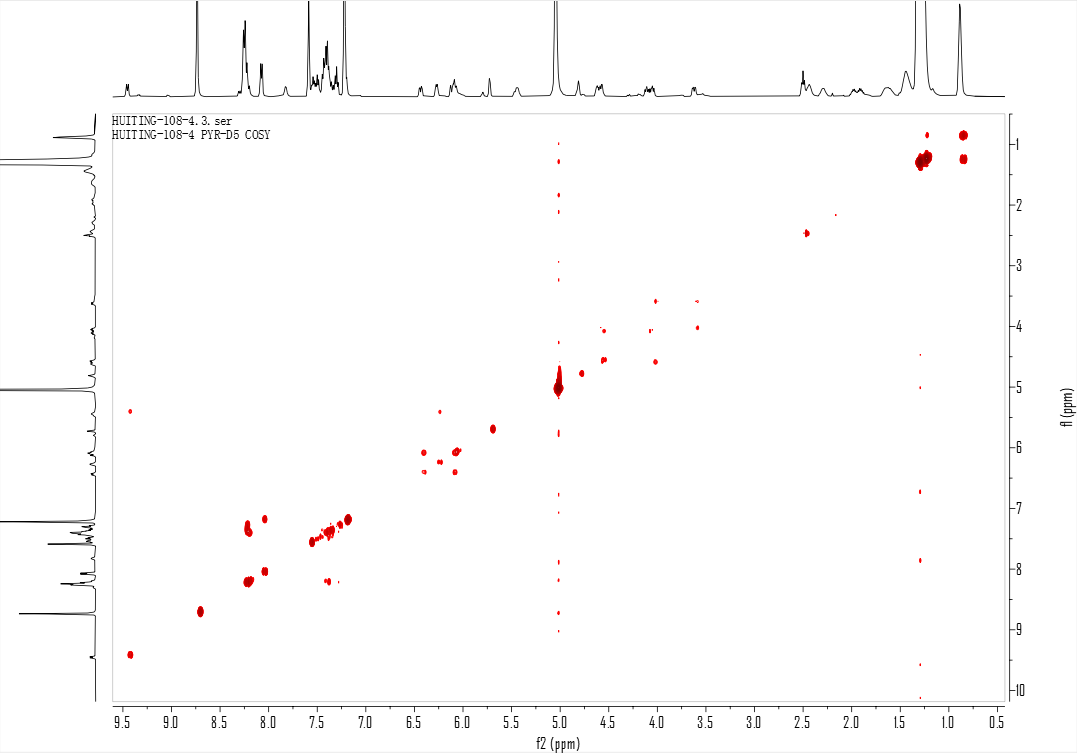
**

**Figure S8.** NMR spectrum of compound (**4**)


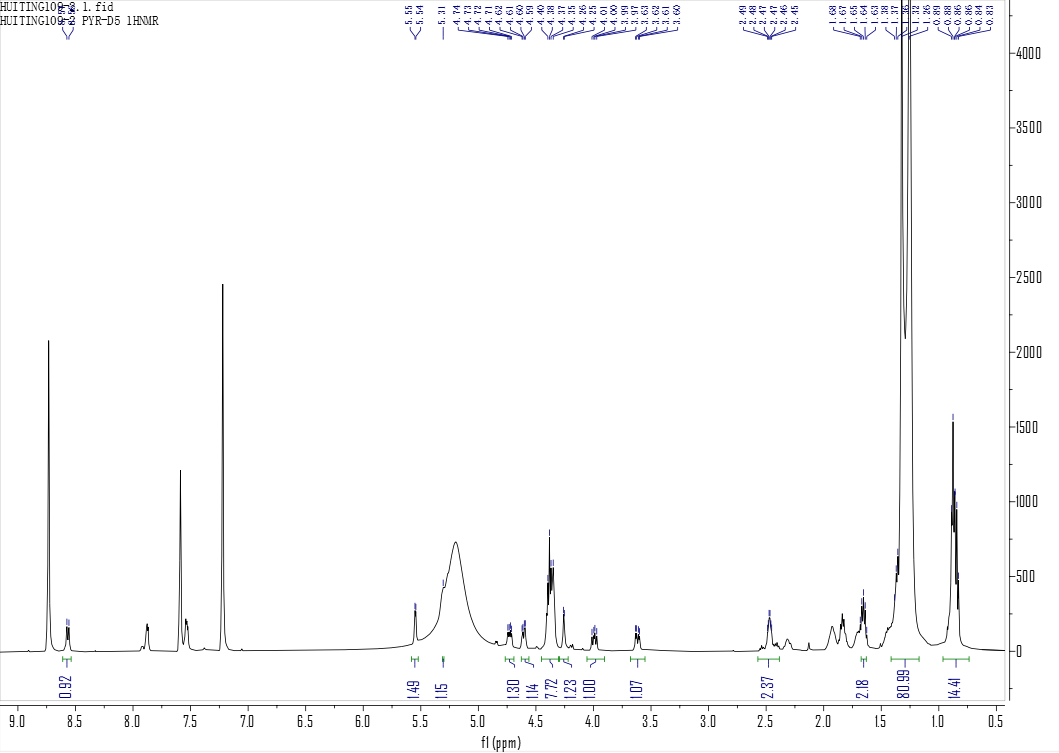


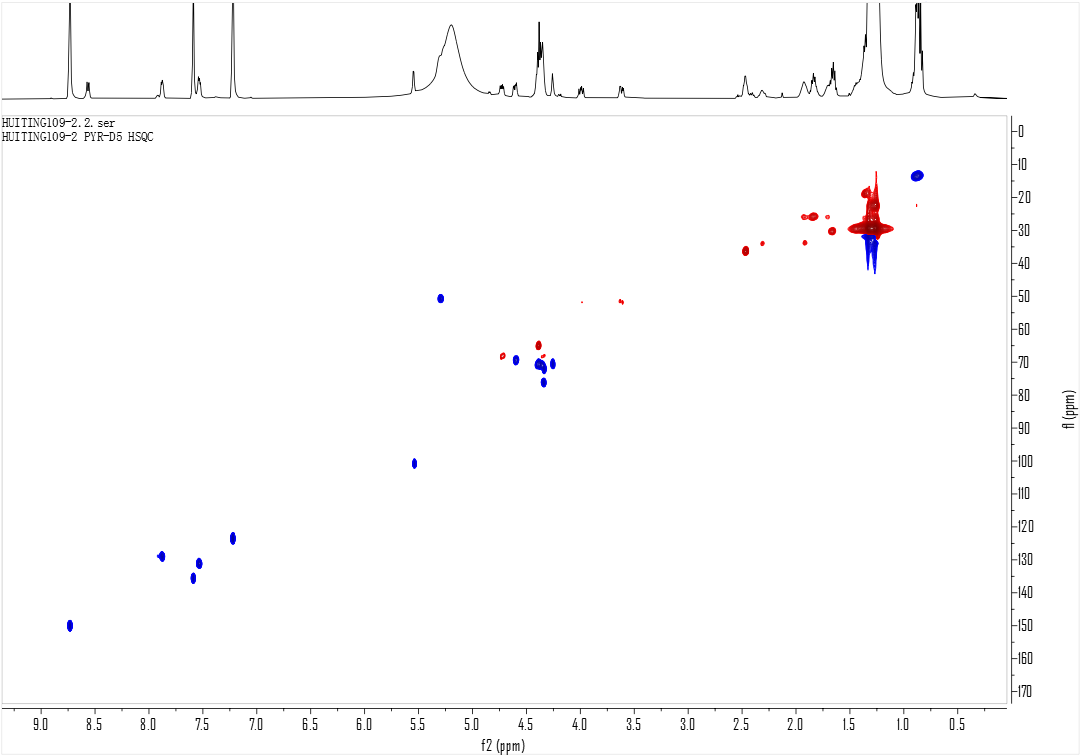


**
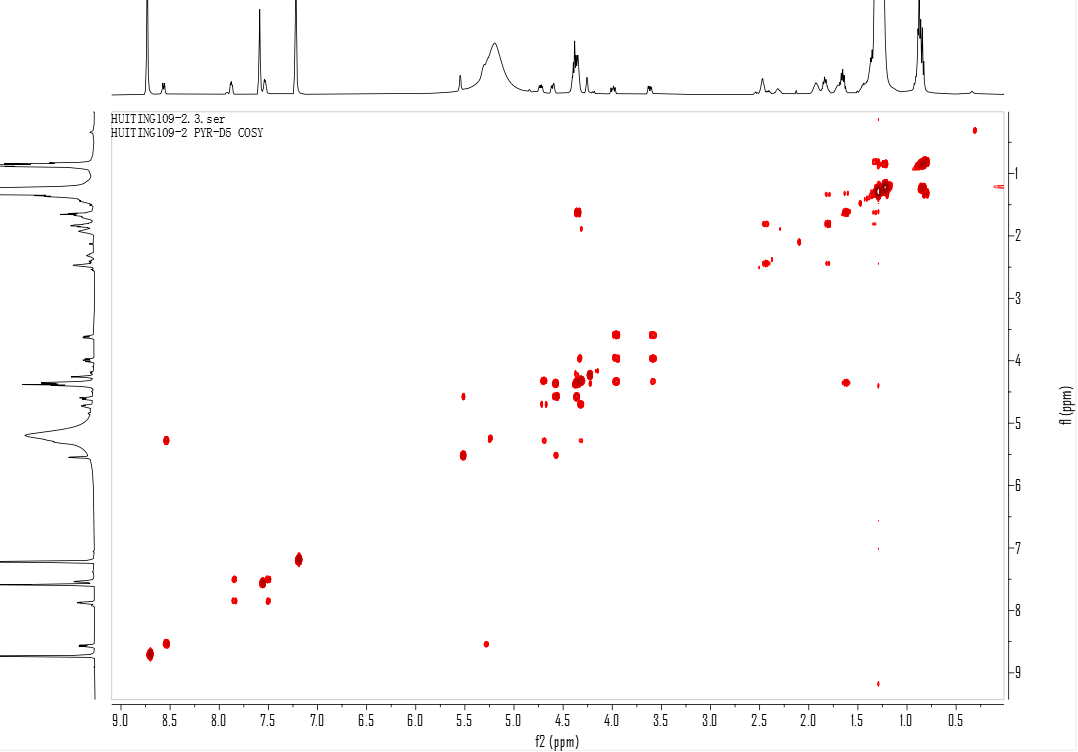
**

**Figure S9.** NMR spectrum of compound (**1**)


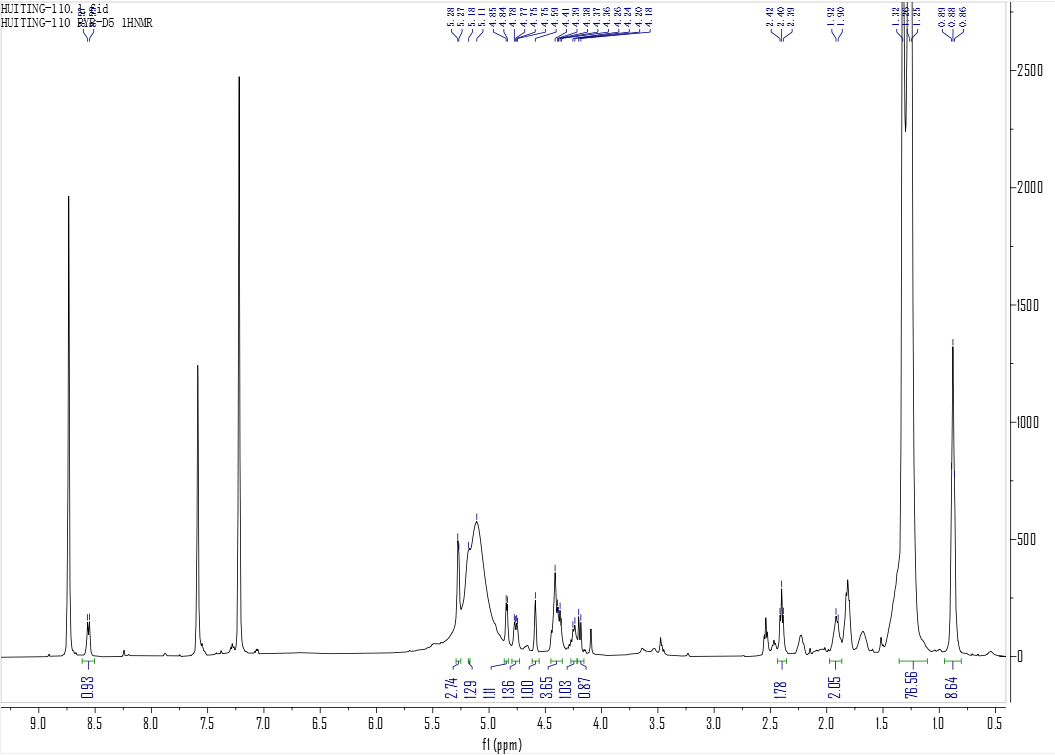


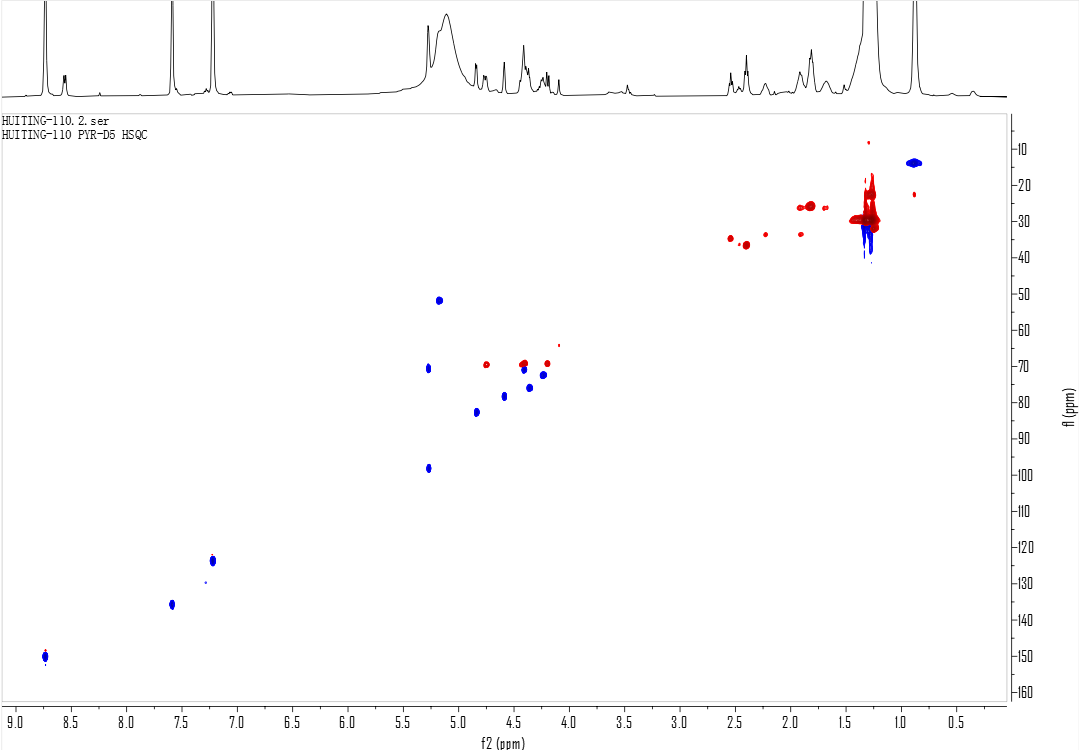


**
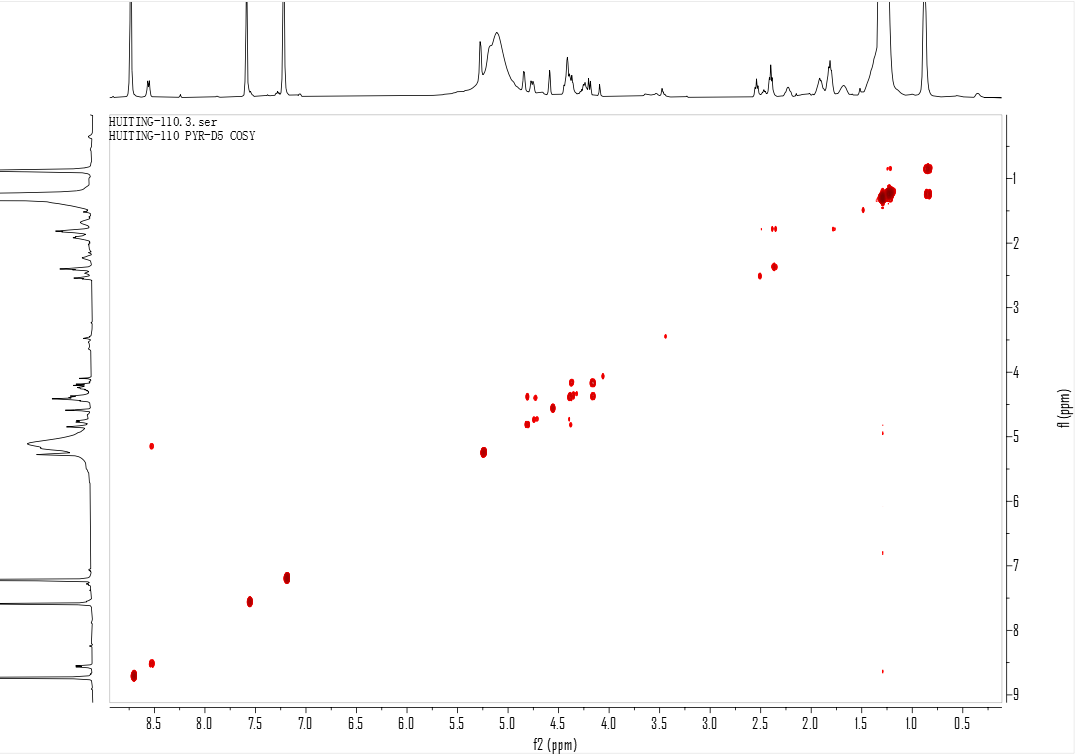
**

**Figure S10.** NMR spectrum of compound (**2**)


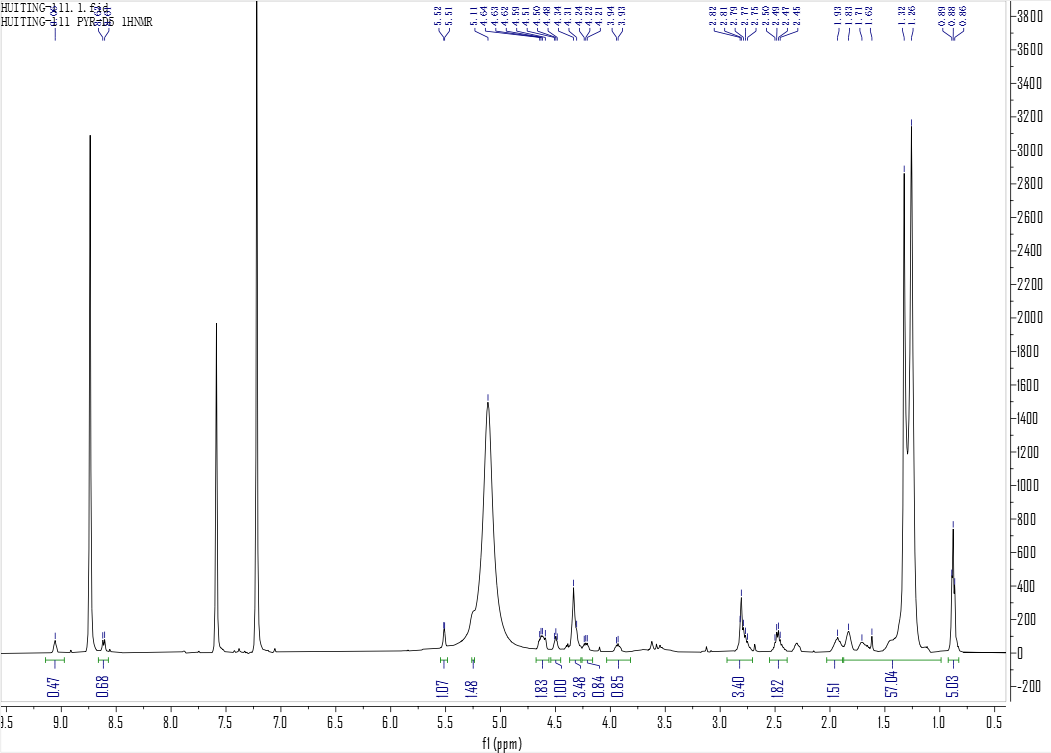


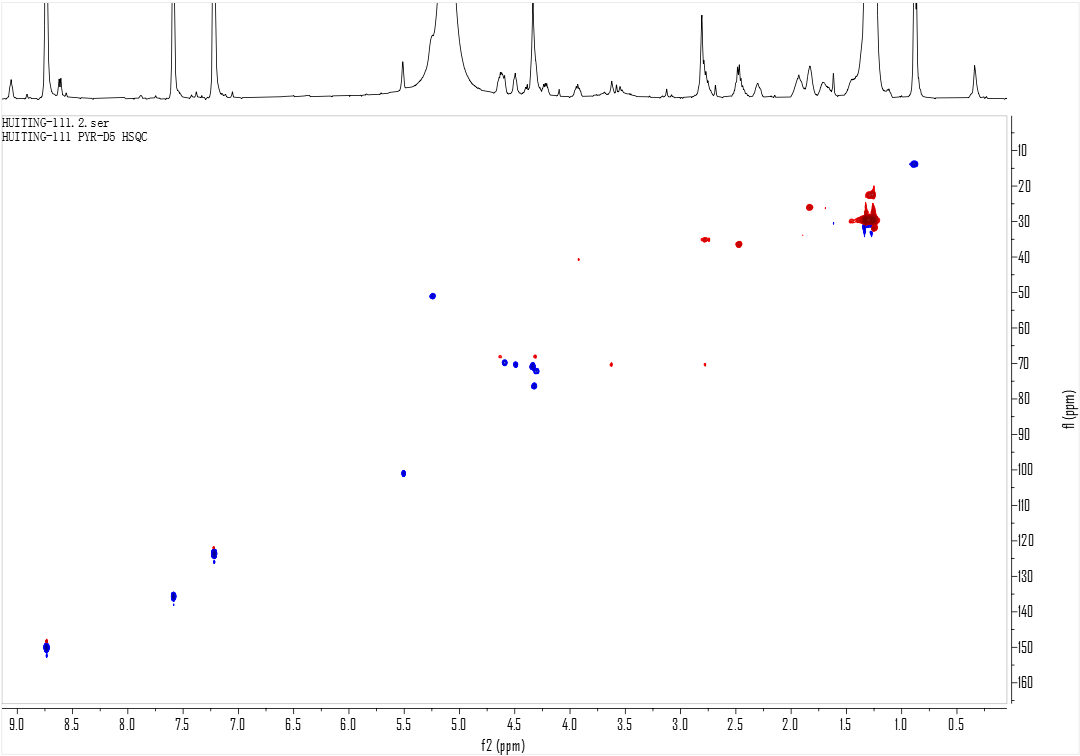


**
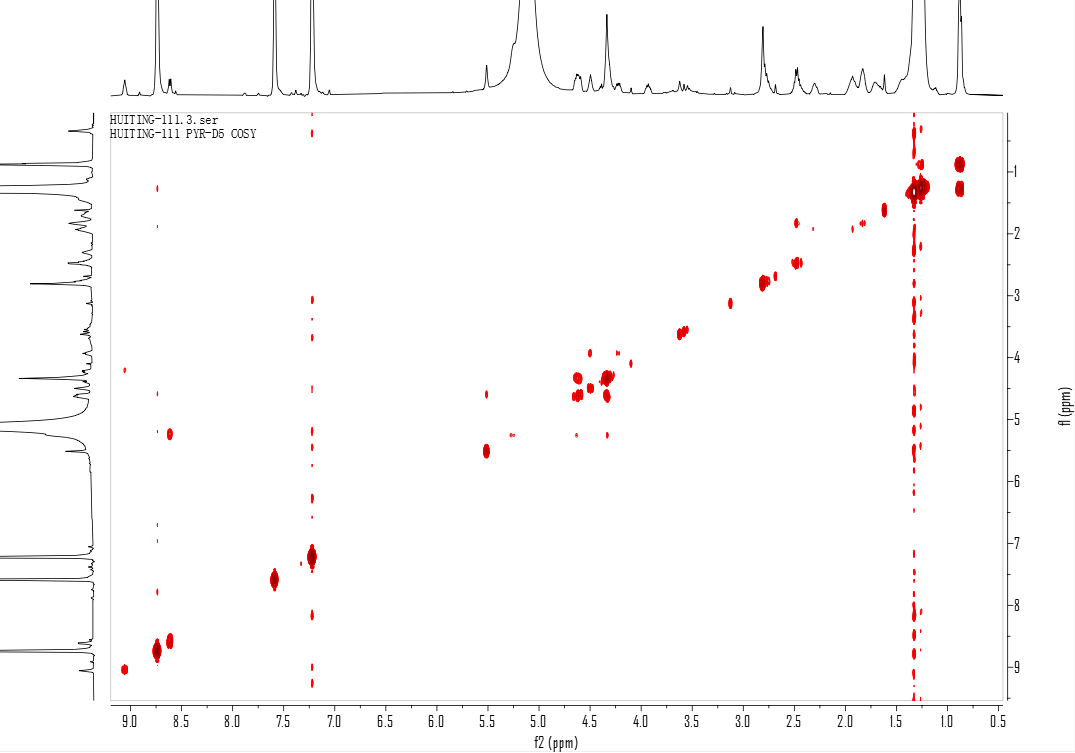
**

**Figure S11.** NMR spectrum of compound (**3**)
